# Acknowledgement to Reviewers of *Materials* in 2018

**DOI:** 10.3390/ma12010194

**Published:** 2019-01-08

**Authors:** Materials Editorial Office

**Affiliations:** MDPI, St. Alban-Anlage 66, 4052 Basel, Switzerland; materials@mdpi.com

Rigorous peer-review is the corner-stone of high-quality academic publishing. The editorial team greatly appreciates the reviewers who contributed their knowledge and expertise to the journal’s editorial process over the past 12 months. In 2018, a total of 2641 papers were published in the journal, with a median time to first decision of 13 days and a median time to publication of 33 days. The editors would like to express their sincere gratitude to the following reviewers for their cooperation and dedication in 2018:

Abadal, SergiLiu, FeiAbali, B. EmekLiu, Fwu-HsingAbbasi, HoomanLiu, Han YinAbbou, SofyaneLiu, JingfeiAbdelghani, AssafLiu, KunAbdel-Mohsen, A.M.Liu, PeiAbdel-Mohti, AhmedLiu, QiangAbdel-Motaleb, Ibrahim M.Liu, QingfanAbdi, FrankLiu, RenAbdollahnejad, ZahraLiu, RuiAbdollahzadeh Jamalabadi, Mohammad YaghoubLiu, SongpoAbdul Samad, YarjanLiu, TianyuAbdul-Aziz, AliLiu, Xian HuAbdullah, Oday IbraheemLiu, XifengAbedini, ArminLiu, XuqingAbejón, RicardoLiu, YaolongAbendroth, BarbaraLiu, YongAbisset-Chavanne, EmmanuelleLiu, YuanAbou Neel, Ensanya A.Liu, Yu-JingAbramovich, HaimLiu, ZhendongAbrate, SergeLiu, ZhiAbuabed, Alaeddin S. A.Lizundia, ErlantzAbuillan, WasimLlosa Tanco, Margot A.Abuin, GracielaLo Conte, AntoniettaAbushammala, HatemLo Frano, RosaAccardo, GraziaLo, Huang-MuAchilias, Dimitris S.Lo, KinhoAchinivu, EzinneLo, Kuang YaoAcosta, MatiasLöbmann, PeerAcuna, GuillermoLoeser, WolfgangAdachi, SadaoLogé, Roland E.Adachi, ShinichiroLogsdail, Andrew J.Adamiec, JanuszLoidl, AloisAddicoat, MatthewLolla, DineshAddison, OwenLombardi, MariangelaAdhikari, RajdeepLonardo, AmedeoAdlhart, ChristianLong, Gary J.Adnan Raheel Shah, SyyedLongana, Marco L.Adrian, HenrykLonghi, MariangelaAfanasiev, P.Longstaffe, James G.Afsarimanesh, NasrinLópez De Lacalle, Luis N.Afshar, ArashLópez De Lacalle, Luis NorbertoAfshinnia, KavehLopez, FrancescoAghazada, SadigLopez, Gabriel AlejandroAgonafir, Mesfin BelaynehLópez-Lorente, Ángela InmaculadaAgrahari, VivekLópez-Ortega, AlbertoAgrawal, Kumar VaroonLopez-Ramon, Maria VictoriaAgrawal, RichaLoprencipe, GiuseppeAgrela, FranciscoLorenzi, AndreaÁgueda, Vicente I.Lorenzo, MiguelAguiló-Aguayo, NoemíLorusso, MassimoAguirre, NestorLos, PrzemyslawAgustin, DominiqueŁosiewicz, BożenaAhmad, Hafiz WaqarLostao, RubenAhmadi, FarzadLou, YanshanAhmadivand, ArashLourenço, MirthaAhmed, EhabLouzguine, Dmitri V.Ahmed, MaryaLova, PaolaAhmed, SnoberLove, Corey TAhmmed, K.M. TanvirLow, It-Meng (Jim)Ahn, ByungminLöwe, HenningAhn, JinsooLowe, ShehanAhn, KollbeLowe, TerryAhonen, MerjaLozac’h, MickaëlAikawa, ShinyaLozano-Guerrero, Antonio JoséAimi, AkihisaLu, LiqiangAimi, JunkoLu, MingyangAinsworth, Richard I.Lu, YaoAkanbi, TaiwoLub, JohanAkazawa, HouseiLube, TanjaAkhavan Niaki, FarbodLucarelli, CarloAkita, SadanoriLucas, DonaldAkitsu, TetsuyaLucas, RomainAkiyama, EijiLuciani, GiuseppinaAksel, HacerŁuczak, SergiuszAl Dushaishi, MohammedLufrano, FrancescoAlam, Md. MehboobLuhrs, Claudia C.Al-Ameri, TalibLui, EricAl-Anssari, SarmadLuis, Felipe SeséAlapan, YunusLuisetto,, IgorAlarco, JoseLukac, PavelAlastalo, AriLukashev, PavelAlat, EceŁukasik, RafałAlba, MariaLukaszkowicz, KrzysztofAlbert, MachokeLukinavicius, GrazvydasAlberto, NéliaLukomska-Szymanska, MonikaAlbizuri, JosebaŁukowski, PawełAlcock, BenLukyanov, PavelAlconero, Patricia LuisLund, AnjaAlcoutlabi, MatazLungu, ClaudiuAldakov, DmitryLuo, QuanshunAldazabal, JavierLuo, RongcongAldwell, BarryLuong, Dzung DinhAlecci, ValerioLuongo, AngeloAlegre, CinthiaLutsyk, PetroAleksandrova, MariyaLuty, TadeuszAlessandro, Dell’EraLützenkirchen, JohannesAlessi, RobertoLützenkirchen-Hecht, DirkAlexander, CameronLuukkonen, TeroAlexandropoulos, Dimitris I.Luzi, FrancescaAlexandrov, SergeiLy, RamatouAlexiou, ChristophLyons, JohnAlexopoulos, AthanassiosŁyszkowski, R.Alfano, BrigidaŁyszkowski, RadosławAlhalawani, AdelM. Karimi, MohammadAlhussein, AkramMa, AnxinAli, Dr. Md. AzaharMa, DuanchengAli, SamimMa, HonganAlikin, DenisMa, JiajuAlipanah, MortezaMa, XiaoliAlique, DavidMaalouf, AzarAljabali, AlaaMaamoun, Ahmed H.Aljazaeri, Zena R.Maazoun, AzerAlleg, SafiaMabbott, SamuelAlMangour, BandarMacak, Jan M.Almeida, AmeliaMaccaferri, NicolòAlmeida, HenriqueMacdonald, NiallAlonso, JavierMacdonald, ThomasAlsagabi, SultanMachrafi, HatimAltan, M. CengizMacKenzie, Kenneth J.D.Altmann, BrigitteMackinnon, Ian D. R.Altstädt, VolkerMadan, DeepaAlvarado Chavarin, CarlosMadden, DavidAlvarez, J. IgancioMaeder, XavierAlvarez, LauraMagalhães, Fernão D.Álvarez, MarMagan-Fernandez, AntonioAlvarez-Alonso, PabloMagasinski, AlexandreAlves, ElianaMagier, MariuszAlves, MartaMagkoev, TamerlanAlzina, FrancescMagna, GabrieleAmanov, AuezhanMagne, SylvainAmendola, RobertaMagyari, KlaraAmin, M. JunaidMahapatra, Manoj K.Amiri, AliMahapatra, MausumiAmoako, KagyaMahata, Manoj KumarAmreddy, NarsireddyMaher, PamelaAmza, CatalinMahesh, SachithanadamAn, JiaMahian, OmidAnagnostopoulos, Costas A.Mahmoud, AbdelfattahAnanthakrishnan, Soundaram JeevarathinamMaia, Lino M.Anastasiou, AntoniosMaier, GerhardAnastasiou, E.K.Maier, PetraAncuţa Elena, TiucMailoa, JonathanAnders, AndréMaiorana, CarloAnderson, Wayne A.Maireles Torres, PedroAndo, YasutakaMaiz, JonAndreev, Dmitrii E.Majak, JüriAndreoli, EnricoMajewski, LeszekAndreopoulou, Aikaterini K.Majlinger, KornelAndreou, ChrysafisMajor, IanAneziris, OlgaMakarona, E.Angelico, RuggeroMäkelä, Jyrki M.Anggraini, Sri AyuMäki-Arvela, PäiviAngilella, Giuseppe G. N.Maksoud, TalalAngione, Maria DanielaMakvandi, PooyanAngrecka, SabinaMalacrinò, AntoninoAngurel, L.A.Malaiškienė, JurgitaAnicai, LianaMałecki, KrzysztofAnjos, OfeliaMałgorzata, MatusiakAnkhili, AmaleMaliaris, GeorgiosAnnamalai, LeelavathiMalka, DrorAnnamalai, Pratheep KumarMallavia, RicardoAnnanouch, Fatima EzahraMalliakas, ChristosAnnunziata, MarcoMallow, OleAnsari, FarhanMalucelli, GiulioAnsón Casaos, AlejandroMalviya, Kirtiman DeoAntaris, Alexander LeeMamba, GcinaAntipov, Oleg L.Manach, Pierre YvesAntoni, JeromeManakhov, AntonAntonio, ApicellaMancusi, ErasmoAntunes, ElsaMandal, SarthakAnyszka, RafałMandić, VilkoAoh, J.N.Mandracci, PietroAoki, DanManera, Maria GraziaAoni, Rifat AhmmedManfredi, LuigiAoude, HassanMangayil, RahulAoyagi, KentaMangialardi, TeresaAparicio Rebollo, Francisco JavierMangunuru, Hari Prasad ReddyApartsin, EvgenyManickam, NandiniApel, MarkusManjunath, ManuboluAphale, AshishManke, IngoApostoluk, AleksandraMankey, GaryApreutesei, MihaiManolakos, DimitriosAquilano, DinoManrique, JavierArabzadeh, AliMansfield, EdwardAradilla, DavidMansour, RabihAranda, Miguel A.G.Mansouri, AliArany, PraveenMansouri, AmirAraromi, OluwaseunMante, Francis K.Arbenin, Andrei Y.Manthina, VenkataArdelean, Lavinia CosminaMantovani, LucianaArena, FrancescaManuel, Pascual GuillamónArenillas, AnaManukyan, Khachatur V.Arias-Pardilla, JoaquinMao, YunweiAriga, KatsuhikoMarafona, José DuarteArima, HirofumiMaraloiu, Valentin-AdrianArjmand, MohammadMarasso, Simone LuigiArkar, CirilMarcadon, VincentArmentani, EnricoMarcelli, Augusto ClaudioArmentano, IlariaMarcellini, MorenoArmstrong, GlenMarchesan, SilviaArnold, WalterMarchese, GiulioArora, AshishMarcian, PetrArora, HimanshuMarcinkevicius, SauliusArpagaus, CordinMargueron, SamuelArrebola, José CarlosMariani, StefanoArrieta, MarinaMarin, M. LuisaArrigo, RossellaMarinkovic, NebojsaArroyo Martínez, BorjaMarino, GiuseppeArtetxe, BeñatMarkopoulos, Angelos P.Artioli, NancyMarkowska-Szczupak, AgataArtola, GarikoitzMarohnic, TeaArumugam, VeerakumarMarotti, JulianaArunachalam, ShivaramMarović, IvanArvapally, Ravi KumarMarpu, Sreekar BabuArvinte, AdinaMarques, CarlosArya, JayaMarques, ClaudiaAryan, PouriaMarradi, AlessandroArzamendi, GurutzeMarrani, AndreaAsadchy, ViktarMarrocchi, AssuntaAsadi, AmirMarszalek, MartaAsadi, HamedMartella, DanieleAsaee, ZohrehMarthinsen, KnutAseev, V. A.Martín, Antonio E.Asenath-Smith, EmilyMartin, Jose MiguelAsensio, Maria C.Martin, Richard A.Asfin, Ruslan E.Martín-de León, JudithAshley, JonMartinelli, EnzoAshok Kumar, MeiyazhaganMartinelli, PaoloAsmontas, SteponasMartínez-Donaire, Andrés JesúsAspatwar, AshokMartínez-Lillo, JoséAstakhov, Viktor P.Martinez-Paneda, EmilioAstarita, AntonelloMartínez-Pañeda, EmilioAta, SeisukeMartinez-Triguero, JoaquinAtabaev, TimurMartinho, José GasparAtanase, LeonardMartinka, JozefAthanasiou, ChristosMartino, MarcoAtia, HananMartín-Ramos, PabloAtrens, AndrejMartins Amaro, AnaAtsawasuwan, PhimonMartins, Ian JamesAttar, HooyarMartins, Luísa MargaridaAttard, DaphneMartins, RodrigoAttila, BaloghMartins, Rui C.Attri, PankajMartoïa, FlorianAtuchin, Victor V.Márton, KivovicsAubry, SylvieMartone, AlfonsoAuclair, NicolasMaruda, Radosław W.Auffermann, GudrunMarulo, FrancescoAurangzeb, QaziMaruyama, KeiAuvray, XavierMarx, MichaelAuzelle, ThomasMasashi, IshikawaAvaro, JonathanMasazumi, OKIDOAverkiev, NikitaMassella, DanieleAversa, AlbertaMasselli, ClaudiaAvgouropoulos, GeorgeMassera, JonathanAw, KeanMastali, MohammadAwana, Veer P.S.Mastrangeli, MassimoAwd, MustafaMateo, AntonioAyaso, Francisco-JavierMateos, Miguel AngelAydin, AlperMaterer, Nicholas F.Azar, Amin S.Mathew, NithinAzarbayejani, MohammadMatias, VladimirAznárez González, Juan JoséMatikonda, SiddharthBabiarz, BożenaMatis, BruceBacariza, CarmenMatko, VojkoBaccarani, GiorgioMatos, InêsBacciocchi, MicheleMatsubara, MasahikoBachaga, TarekMatsugo, SeiichiBacigalupo, AndreaMatsuki, YohBadilescu, SimonaMATSUMOTO, ArimasaBadin, GualtieroMatsuo, TetsujiBae, ChangdeuckMatsushima, HisayoshiBae, JoonwonMatsushima, YutaBae, SungchulMattelaer, FelixBaeza, Francisco JavierMatula, GrzegorzBagdziunas, GintautasMauer, GeorgBaigorri, RobertoMaunier, CedricBaimova, JuliaMaurice, DavidBaino, FrancescoMauriello, FrancescoBajda, TomaszMautner, AndreasBaker, PaulMavridis, IoannisBalakrishna, Ananya RenukaMay, StevenBalamurugan, Sreelatha S.Mayer, MatejBalan, TudorMayer, ThomasBalart, MariaMayuet, PedroBalasingam, Suresh KannanMazahery, AliBalasubramanian, KarthikMazeika, LiudasBalázsi, CsabaMazurek, GrzegorzBaldo, NicolaMazzotti, MatteoBaldovín, Francisco LópezMcCue, IanBalduzzi, GiuseppeMcHugh, KevinBalintova, MagdalenaMcKenzie, EricaBallesté, Ruben MasMcLean, AlexanderBalloy, DavidMcMillan, Alison J.Balogi, ZsoltMeaud, JulienBaloukas, BillMéausoone, Pierre-JeanBaltakys, KęstutisMederic, PascalBaltriukiene, DaivaMedvecky, LubomirBalzer, FrankMedved, SergejBanach, MarcinMedyński, DanielBandi, VenuMeenashisundaram, Ganesh KumarBandiello, EnricoMegalini, LudovicoBandodkar, Amay J.Megiel, ElżbietaBandura, LidiaMehrpouya, MehrshadBandyopadhyay, SulalitMei, LeiBandyopadhyay, SupriyoMekonnen, Tizazu H.Banerjee, Shib ShankarMele, AndreaBańkowski, WojciechMelianas, ArmantasBañón, LuisMélinon, PatriceBao, YiMelnykowycz, MarkBao, YinyinMelro, António RuiBar, IlanaMen, YongjunBarabás, RékaMenabue, LediBarandiaran, IratiMenapace, CinziaBarany, TamasMendes, JérômeBarbaglio, AliceMeneghetti, GiovanniBarbato, Felicia Carla TizianaMenendez, BeatrizBarbera, JoaquinMeng, F.Q.Barberis, AntonioMenger, FredricBarbetta, AndreaMengucci, PaoloBarbier, A.Mensi, MounirBarbieri, MaurizioMenzemer, CraigBarbillon, GrégoryMerkel, MarkusBarbosa, JoaquimMerola, Simona SilviaBarbot, Jean FrancoisMeroni, DanielaBardella, LorenzoMertas, AnnaBarile, ClaudiaMesarovic, SinisaBarille, RegisMeshi, LouisaBarlage, Douglas W.Messinger, RobertBarman, ManikMessyasz, BeataBarmparis, Georgios D.Mestre, Ana SofíaBarnat-Hunek, DanutaMestre, Ana Sofia DiasBaroutaji, AhmadMetaferia, WondwosenBarra, GiuseppinaMeyer, Jerry R.Barranco, AngelMeza, LucasBarré, OlivierMi, ShengliBarretta, RaffaeleMiccio, FrancescoBarriobero-Vila, PereMichal, CarlBarry, CarolMichalcová, AlenaBarskiy, DanilaMichalska-Domańska, MartaBarszczewska-Rybarek, IzabelaMichelhaugh, Sharon KayBartocci, PietroMicheli, DavideBartolomeo, Antonio DiMichl, Thomas DannyBartonickova, EvaMickelson, Alan R.Bartsch, TimoMiedzińska, DanutaBartuli, CeciliaMigita, SatoshiBaruth, AndrewMignon, ArnBasak, AnupMihara, TsuyoshiBasaran, CemalMija, AliceBasiak, EwelinaMikhaylovskaya, A. V.Basova, TamaraMikhelson, KonstantinBassani, MarcoMiki, HajimeBäßler, RalphMiklaszewski, AndrzejBastos, AntónioMikno, ZygmuntBasu, SaurabhMikolajczyk, TadeuszBattistel, AlbertoMikula, MariánBatule, Bhagwan SahebraoMilanesio, MarcoBaudis, StefanMilčius, DariusBauer, FrancoisMilcovich, GesmiBaumann, Felix W.Miled, AmineBayer, IlkerMilia, EgleBayraktar, EminMiličević, IvanaBeake, BenMiller, ErinBeal, ColineMillithaler, Jean-FrançoisBeall, GaryMills, AndrewBear, Joseph C.Milosevic, MiloradBeard, James D.Milovanovic, BojanBeber, Vinicius C.Miluski, PiotrBeck Jr, George R.Minakshi, ManickamBecker, KathrinMinami, IchiroBecker, ThorstenMinarik, PeterBedia, JorgeMinas, GraçaBedon, ChiaraMinelli, MatteoBeeton, NickMineva, TzonkaBégin, DominiqueMingareev, IlyaBehnood, AliMingler, BernhardBehrens, Bernd-ArnoMingo, AntonioBei, GuopingMiniaci, MarcoBekiaris, GeorgiosMiniewicz, AndrzejBelber, CarolinaMino, LorenzoBełdowski, PiotrMINOHARA, MakotoBelhocine, AliMintcheva, NeliBellayer, SéverineMiranda Salvado, Isabel MargaridaBellini, CostanzoMiranda-Castro, RebecaBellotto, MaurizioMiras, Haralampos N.Belussi, LorenzoMircea, NicoaraBelyakov, AndreyMiroshnichenko, AndreyBelzunce Varela, Francisco JavierMirsayar, MirMiladBencardino, FrancescoMishima, MasanoriBenedetti, AlessandroMishin, OlegBenedetti, IvanoMishra, Pawan KumarBenedict, BarryMissinne, JeroenBenfer, SigridMitchell, Scott G.Bengani, Lokendra KumarMitelea, IonBeni, ValerioMitsuhiro, TerakawaBeniah, GoliathMittal, NiteshBeniak, JurajMiu, DanaBenmokrane, BrahimMiyamoto, HiroyukiBenoit, DenizotMiyamoto, ManabuBenseman, Timothy M.Miyata, NaoyukiBeranoagirre, AitorMiyazaki, ToshikiBerardi, UmbertoMiyoshi, NorioBerardi, Valentino PaoloMizera, AlesBerbecaru, Andrei ConstantinMizsei, JánosBéreaux, YvesMizuno, MasashiBeretta, S.Młynarczykowska, AnnaBerg, SteffenMo, KunBerge, FranzMobed-Miremadi, MaryamBermudo, CarolinaMobili, AlessandraBernal, M.MarMoghaddam, Hesam S.Bernardello, FabioMohamed, AlaaBernardi, SaraMohamed, WalidBernardino, Raul J.Mohammad Karim, AlirezaBernardo, EnricoMohammadi, MohsenBernardo, LuísMohammadiarani, HosseinBernards, MatthewMohammed, AlyaaBernède, J.c.Mohammed, Ghusoon RidhaBernhard, ChristianMohanta, AntaryamiBerski, WiktorMohd-Yasin, FaisalBerteau, Jean-PhilippeMohles, VolkerBerthebaud, DavidMohr, JuergenBertocci, FrancescoMohtadi Bonab, Mohammad AliBertolotti, FedericaMoiseev, SergeyBerton, PaulaMokaya, RobertBertoncello, PaoloMolazemhosseini, AlirezaBertsch, ArnaudMolenda, JaninaBeschkov, VenkoMolina, C.B.Besozzi, EdoardoMolino, PaulBessa, LucindaMolla, AshrafulBhandari, KhagendraMollenhauer, KonradBhang, Suk HoMombelli, DavideBhattacharjee, SonaliMomeni, MojtabaBhattacharya, SumitMonchau, FrancineBhosale, SheshanathMondal, KunalBhowmick, SukantaMondal, SudipBi, GuijunMondloch, JoeBiałobrzeska, BeataMonetta, TullioBiasi, PierdomenicoMonková, KatarínaBibicu, IonMonroe, Mary Beth BrowningBielinski, DariuszMonsalve, AlbertoBieliński, Dariusz M.Montanari, RobertoBieniaś, JarosławMontealegre-Melendez, IsabelBieńkowski, AdamMontero, FranciscoBiernasiuk, AnnaMontero-Chacón, FranciscoBiernat, JanMontes Ruiz-cabello, Francisco Javier MontesBiesuz, MattiaMontgomery, JasonBigi, AdrianaMontgomery, M.Bihamta, RezaMonti, AlessioBilal, Osama R.Montuori, RosarioBílek, VlastimilMonzón, MarioBirch, BrianMonzón, Mario D.Birkett, MartinMoon, Byung-MoonBirleanu, CorinaMoon, Doo KyungBiswal, Bishnu PrasadMoon, Hong ChulBiswanath, DuttaMoon, Jong-yunBiswas, SoumavaMoon, JuhyukBjörkman, TorbjörnMoore, Axel C.Blakley, Sean M.Moradi, BaharehBlanc, D.Morales-González, José AntonioBlanco, IgnazioMorales-Guio, CarlosBlaser, MarkMorales-Palma, DomingoBlock, Jean-ClaudeMorasch, AlexanderBloh, Jonathan Z.Moreno Pineda, EufemioBludov, YuliyMoreno, PabloBlunt, LiamMoreno-Atanasio, RobertoBobinger, MarcoMoreno-Maroto, José ManuelBocci, EdoardoMorgan, AlexanderBoccia, Antonella CaterinaMorgiel, JerzyBochenek, DariuszMori, AkihisaBockowski, MichalMori, HideharuBogdanowicz, Krzysztof ArturMori, KohsukeBogni, AlessiaMorikawa, AtsushiBoichot, R.Morimoto, YuBolander, JohnMorin, LéoBollella, PaoloMoritz, MichalBolotov, LeonidMorley, Nicola ABombac, DavidMoron, CarlosBon, VolodymyrMoroz, AdamBonani, WalterMorreale, MarcoBondar, DaliMorri, AlessandroBondarev, Andrey V.Morrow, BenjaminBonduelle, Colin V.Morrow, RyanBonek, MirosławMortazavi, BohayraBonelli, FrancescoMoseke, ClausBong, Hyuk JongMosey, StephenBontempi, ElzaMoskal, GrzegorzBontempi, NicolòMosleh, Ahmed O.Borchers, C.Mostafa, AhmadBorcia, GMostoni, S.Bordeenithikasem, PunnathatMotra, Hem BahadurBordia, Rajendra K.Motyka, MaciejBorgesen, PeterMotzki, PaulBorhani, TohidMouchet, SébastienBorkowski, AndrzejMoulick, AmitavaBorkowski, LeszekMoura, Maria J.Bormashenko, EdwardMourdikoudis, StefanosBorodachenkova, MarinaMourtas, SpyridonBorodianskiy, KonstantinMoussaoui, YounesBoronat Vitoria, TeodomiroMoutou Pitti, RostandBorri, ClaudiaMrówczyński, RadosławBorsacchi, SilviaMróz, WaldemarBorzabadi-Farahani, AliMrukiewicz, MateuszBos, Freek P.Mu’azu, Nuhu DalhatBosia, FedericoMuc, AleksanderBossis, GeorgesMucha, JacekBostan, IonelMuciaccia, GiovanniBotticelli, DanieleMuench, FalkBotto, DanieleMuguruma, HitoshiBoumerzoug, ZakariaMukai, KeisukeBourillot, EricMüller, BettinaBouropoulos, NikolaosMüller, GerhardBoz, IlkerMULLER, RobertBranco, RicardoMun, JiwonBrand, AlexanderMünch, AlexanderBrandt, MilanMuniz-Miranda, FrancescoBranício, Paulo SergioMuniz-Miranda, MaurizioBrant, Jonathan A.Muñoz, AsunciónBrantley, William A.Muñoz, JoseBravo, Antonio GilMuñoz-Batista, Mario JesúsBredol, MichaelMunoz-Bonilla, AlexandraBrestic, MarianMunteanu, Bogdanel SilvestruBreveglieri, MatteoMunz, Richard J.Brezesinski, TorstenMura, AndreaBrillard, AlainMurakami, NaoyaBritton, T. BenMurakami, TeruoBrnić, JosipMurcia-López, SebastiánBroda, JanMuschiol, JanBromme, DieterMusmarra, DinoBrosseau, ChristianMust, IndrekBrostow, WitoldMuszynski, LarryBrouzgou, AngelikiMuthaiyan, ArunachalamBrown, Judith A.Mutyala, KalyanBrown, Trevor C.Myers, OliverBruckner, GudrunMystkowska, JoannaBrühwiler, DominikNa, OkpinBrunella, ValentinaNaderi Kalali, EhsanBrunesi, EmanueleNadimpalli, Siva P.V.Brunetto, Priscilla S.Nadja, RohrBrunner, AndreasNadolna, JoannaBrutti, SergioNadolny, KrzysztofBryan, EigenbrodtNadolny, ZbigniewBrychcy-Rajska, EwaNag, ManojBrytan, ZbigniewNagai, HirokiBrzeziński, MarekNagai, KoheiBuccheri, Maria-AntoniettaNagatani, NaokiBuccolieri, AlessandroNaghavi, S. ShahabBuchet, ReneNagy, EndreBuchheit, Rudolph G.Naha, Pratap C.Bucinskas, VytautasNahar, LamiaBuckman, JimNahata, SudhanshuBugaev, AramNair, Arun K.Bui, Tinh QuocNair, DevathaBuj, IreneNair, RemyaBulgariu, LauraNair, Sriramya D.Bunaziv, IvanNajafi, HosseinBuonocore, FrancescoNajarian, SiamakBuratti, CinziaNajm, Husam S.Burja, JakaNak Cheon, JeongBurlacu, AlexandrinaNakagawa, YoshinaoBurmistrov, I.N.Nakamura, TatsuyaBurpo, Fred J.Nakazawa, YasumotoBurrows, NathanNakielski, PawelBury, WojciechNam, JeongsooBusquets, Maria AntòniaNam, Ki-WooBussolati, OvidioNanba, TetsuyaBustillo, JulienNand, AshveenButler, JohnNanda, Kamala KantaButler, LesNandwana, PeeyushButt, JavaidNanev, ChristoBuzuk, MarijoNano, AdelaBychanok, DzmitryNaoki, KawazoeCaballero, AlvaroNaplocha, KrzysztofCaballero, OlgaNara, HirokiCabanelas, Juan CarlosNarayanan, GaneshCabedo, LuisNarciso, JavierCabeza Simo, Marta MariaNarita, FumioCabot, Pere-LluísNaser, M. Z.Cabrales, LuisNastasa, CristinaCabrini, MarinaNatarajan, Thillai SivakumarCaciolli, AntonioNattestad, AndrewCadatal-Raduban, MarilouNaumann, StefanCaddeo, ClaudiaNaumova, Ella A.Caggiano, AntonioNavarro Pedreño, JoseCai, FeiNavarro, CarlosCai, FengNavarro, María VictoriaCaiazzo, FabriziaNavarro, PabloCalà Lesina, AntoninoNavau, CarlesCalabrese, LuigiNavío, J.A.Calaon, MatteoNazarov, Andrej P.Calì, MicheleNazir, Mian HammadCalka, AndrzejNeagoe, MirceaCalvete, MarioNečas, JanCalvo Guirado, José LuisNedeljkovic, MarijaCalvo-Dahlborg, MoniqueNee, Matthew J.Calvo-Guirado, José LuisNegahban, MehrdadCamacho, Ana MaríaNegrete, GeorgeCamarillo, RafaelNegulescu, IoanCambiasso, JavierNeimitz, AndrzejCamblor, Miguel A.Nelson, Fred R.T.Cameron, JosephNematollahi, BehzadCamilli, LucaNemes, Norbert M.Caminero, M.A.Nemnes, George AlexandruCamões, AiresNemoto, NobukatsuCampana, RaffaellaNeri, PaoloCampanella, LuigiNeruda, MarekCampbell, JohnNeto, Diogo M.ÇAMURLU, H. ErdemNeuenschwander, JuergCandolfi, ChristopheNeufurth, MeikCaneschi, AndreaNeumeister, PeterCangialosi, DanieleNeves, RuiCannone Falchetto, AugustoNevshupa, Roman ACanpolat, CetinNg, AlphonsusCantera, M AsunNg, AndyCantero Guisández, José LuisNguyen, Ba Thuy LinhCantisani, GiuseppeNguyen, Nhat TruongCao, JianyunNguyen, TuyenCaparrotta, StefaniaNguyen, Van KhanhČapek, JanNian, QiongCapitão, SilvinoNicholson, John W.Capoccia, MassimoNicolau, AnaCapsoni, DorettaNicoletta, Fiore PasqualeCaputo, Gregory A.Nien, Yung-TangCarabineiro, SoniaNiewa, RainerCarabineiro, SóniaNijmeijer, ArianCárcel-Carrasco, Francisco JavierNikas, George K.Carette, JérômeNikhare, ChetanCarlen, Edwin T.Nikkhah-Moshaie, RoozbehCarlino, ElvioNiknam, AlborzCarlone, PierpaoloNikolaidis, Th.N.Carlos, García NúñezNikolic, MijoCarmagnola, IreneNikumb, SuwasCarneiro, Olga SousaNimbalkar, SanjayCarnevale, GianlucaNir, OdedCarosio, FedericoNishida, NaokiCarou, DiegoNishihara, HirotomoCarranza, FranciscoNishihara, MasamichiCarrasco, SergioNishikitani, YoshinoriCarrera-Gallissà, EnricNishimoto, YoshioCarty, William M.Nistor, CristinaCasalino, GiuseppeNitschke, MirkoCascardi, AlessioNitulescu, George MihaiCasey, SeanNiu, ChangningCaspi, YaronNobile, LucioCastagnola, ValentinaNocivin, AnnaCastaldo, RacheleNoguchi, HiroshiCastanheiro, JoséNohe, AnjaCastaño, OscarNommeots-Nomm, AmyCastedo, RicardoNoor, NuruzzamanCastell, PereNoordermeer, JacquesCastellino, MicaelaNordhagen, HåkonCastñeiras, AlfonsoNorouzzadeh, PayamCastro, AndreNosoudi, NasimCastrucci, PaolaNotbohm, HolgerCataldi, PietroNothdurft, FrankCatizzone, EnricoNouranian, SasanCattaneo, SaraNovak, IgorCattoën, XavierNovák, PavelCauwe, MaartenNóvoa-Muñoz, Juan CarlosCavallaro, GiuseppeNowak, PawelCavicchi, KevinNowak, WojciechCazacu, EmilNowicki, MichałCech, VladimirNuić, IvonaCelano, UmbertoNunes, Carla D.Celikin, MertNunes, DanielaCellini, FilippoNussbaumer, AlainCerbu, CameliaNygren, Kelly ElizabethCerrato, GiuseppinaNypelo, TiinaCerri, EmanuelaNypelö, TiinaCerro-Prada, ElenaO’Mahony, ConorCesano, FedericoObbard, Edward G.Cetin, Arif EnginObraztsov, Alexander N.Cha, Young-JinObrosov, AlekseiChai, Kyu YunOchi, MasayukiChaim, RachmanOchowiak, MarekChalal, HocineOda, YukariChalioris, ConstantinOehring, M.Chamonine, MikhailOesterschulze, EgbertChampagne, Victor KennethO’Flaherty, FinChandasana, HardikOgam, ErickChander, HarishOgawa, AkikoChang, Chia-ChenOgawa, ShinpeiChang, Ching-yaoOgino, HirakuChang, JianminOgino, ToshioChang, Sheng-PoOgorek, RafalChang, Te-WeiOgrodnik, PawełChang, Wei-JenOh, Daniel S.Chantana, JakapanOh, Dongyeop X.Chaouch, MounirOh, HyunchulCharilaou, MichalisOh, Min-WookCharles, YannOh, Sang-KeunCharlton, ClivelOhmagari, ShinyaCharmet, Andrea PietropolliOhno, SeigoCharpagne, Marie-AgatheOhta, HiromichiCharvatova, HanaOjovan, MichaelChass, GregoryOkada, NarihitoChateigner, DanielOkayasu, MitsuhiroChathoth, Suresh MavilaOkesola, BabatundeChatterjee, SabornieOkitsu, YoshitakaChaturvedi, PracheeOkoronkwo, MondayChatzinikolaidou, MariaOkoshi, MasayukiChauhan, MoniOkrasa, MałgorzataChauhan, MunishOksiuta, ZbigniewChaunsali, PiyushOkubo, MasayoshiChava, Rama KrishnaOkuda, TetsujiChavez-Angel, EmigdioOkulov, IlyaChavoshi, Saeed ZareOkunkova, AnnaChecchetto, RiccardoOlewnik-Kruszkowska, EwaCHEIKH, KHADIJA ELOliva, LeoneChen, BinQiangOliva, PietroChen, ChiOliveira, GustavoChen, ChunhuiOliveira, João PedroChen, Dyi-ChengOliver-Ortega, HelenaChen, FangOlmos, DaniaChen, HailongOlofinjana, AyodeleChen, HouyangOlsson, RickardChen, JiahaoOluwafunmilayo, JosephChen, JinhuaOnbasli, M. C.Chen, JunO’Neil, GlenChen, Jung-HsuanOniga, Smaranda DafinaChen, LiangOno, KanjiChen, Lung-ChienOptasanu, VirgilChen, MingshengOrbulov, ImreChen, Po-YuOreshonkov, AleksandrChen, Roland K.Örnek, CemChen, ShujianOrrego, SantiagoChen, ShuoOrtega, Eugenio VelascoChen, Shyi-MingOrtega, José MarcosChen, Tai-ChengOrtega, ZaidaChen, Tao-HsingOrtega-Casanova, JoaquínChen, TianyiOrtelli, SimonaChen, Xiao-BoOrtenzi, Marco A.Chen, Yuan-TsungOsman, AhmedChen, Yu-ChihOśmiałowski, BorysChen, ZhangweiOstasevicius, VytautasChen, Zhi-GangOstendorf, AndreasCheng, Chiang-hoOstoja-Starzewski, MartinCheng, Chih-ChiaOstrowski, KrzysztofCheng, Chun-HuOterkus, ErkanCheng, Fong-YuOtero-Espinar, Francisco JavierCheng, GuangmingOtmačić Ćurković, HelenaCheong, In WooOtsuki, AkiraChereddy, Kiran KumarOtsuki, JoeCherevan, AlexeyOuerghi, AbdelkarimCheruzel, LionelOugier-Simonin, AudreyChesneau, XavierOuyang, LiangqiCheung, Angus K.F.Ovchinnikov, AlexanderChia, Win-LongOyedele, AkinolaChiang, Cheng-TienOzbakkaloglu, TogayChiang, Yu-ChunOzen, Mehmet OzgunChiariotti, PaoloOzer, EkinChien, Feng-TsoP. C. Duarte, AntónioChiessi, EsterPablos, CristinaChikamatsu, AkiraPachauri, VivekChikan, ViktorPacheco-Peña, VictorChinniah, YuvinPacheco-Torgal, FernandoChiu, Neng-HsinPaci, MaurizioChiulan, IoanaPadalkar, SonalChizhik, AlexanderPadhy, Girish KumaChladek, GrzegorzPadilla, JavierChmielarz, PawełPadmanaban, RamasamyChmielowski, KrzysztofPadmanabhan, JagannathCho, ChungyeonPaduch, RomanCho, In SunPaeglitis, AinarsCho, Jae UngPagan, DarrenCho, Jae-HyungPagano, StefanoCho, Jong-RaePahlevani, FarshidCho, KeunheePainter, RogerCho, Kuk YoungPaiva, Jose MarioCho, LawrencePakdel, EsfandiarCho, Sung-JinPalanisamy, SelvakumarCho, Young-RaePaleu, ViorelChoi, HyoungPalinko, IstvanChoi, Jae-HongPalka, NorbertChoi, Myong YongPallaoro, AlessiaChoi, SamjinPalma, Luca DiChomicz-Kowalska, AnnaPalumbo, DavideChorazy, SzymonPalumbo, GaetanoChorzepa, Mi G.Pamies, RamonChoudhary, Kumari SonalPAN, Huang HsingChoudhury, SoumyadipPan, QinChowdhury, PiyasPan, Wei-PingChráska, TomášPan, ZengxiChrist, Hans-JuergenPanagopoulos, C. N.Christofidou, Katerina A.Panaro, Maria AntoniettaChristoforides, EliasPandey, GauravChristos, KordulisPandey, SudipChristova, DarinkaPandey, TribhuwanChroneos, AlexanderPanek, RafalChronopoulos, DimitriosPang, YoonsooChruściel, Jerzy J.Pangallo, DomenicoChu, Chun-HungPani, DaniloChun, Dong HyunPanich, Alexander MChung, Sang-YeopPanin, SergeyChurchill, David G.Panjwani, DeepChuryumov, AlexanderPannier, YannickCicala, GianlucaPanomsuwan, GasiditCicciù, MarcoPanowicz, RobertCicconi, PaoloPantano, MariaCicek, KenanPantazopoulos, GeorgeCicero, SergioPaolone, AnnalisaCiesielski, MichaelPapadakis, RaffaelloCieslak, JakubPapadimitriou, VassilikiCieslar, MiroslavPapaefthymiou, SpyrosCifuentes, HectorPapageorgiou, George Z.Cîmpean, AnişoaraPapoulis, DimitriosCinelli, PatriziaPappalardo, Carmine MariaCinteza, Ludmila OtiliaPappalettera, GiovanniCirujano, FranciscoParajuli, DurgaCiuffini, Andrea FrancescoPáramo-García, UlisesCIUTINA, AdrianParedes, José ManuelČizmić, MirtaParedes-Madrid, LeonelClaramunt, JosepParfenov, EvgenyClarke, Ronald JamesParigger, ChristianClaßen, MartinParisi, CristianClayton, John D.Parisi, FilippoClemens, OliverPark, Chul-hoClement, PierrickPark, Dong HyukClimente-Alarcon, VicentePark, JaehoonCoakley, EoinPark, Jang MinCoco, SilverioPark, Jang-UngCodetta-Raiteri, DanielePark, JeyoungCoduri, MauroPark, Ji-SangCoelho, LuísPark, JoontaekCoffetti, DennyPark, Jun-BeomCohen, EstellePark, Jun-HongCola, AdrianoPark, Jun-SangColajanni, PieroPark, Kang HyunColangelo, FrancescoPark, Kwi-IlColangelo, GianpieroPark, NokeunColas, GuillaumePark, SangkiColeman, AnthonyPark, Seong JinColeman, NicholaPark, Shin-YoungCollado, DanielPark, Sol-MoiCollin, MatthewPark, Sun HongCollini, LucaPark, Sung-KyuCollins, Maurice N.Park, WansuColmenares, Juan CarlosPark, You-JinColombi, PierluigiParker, JonathanColorado, HenryParker, Stewart F.Comet, MarcParkes, MariaComuzzi, ClaraParkinson, DilworthConcilio, AntonioPartovi-Nia, RachelConcu, GiovannaPascu, AlexandruConnolly, JamesPascual, LauraConstantin, LucianPasetto, MarcoConterosito, EleonoraPasini, DarioContessi Negrini, NicolaPasinszki, TiborCooke, MichaelPasquale, Claudio DeCooke, MikePasti, LuisaCools, PieterPastore, ChristopherCooney, RalphPastore, RobertoCooper, DanielPastorek, FilipCooper, Ryan C.Paszkiewicz, SandraCoppari, FedericaPatel, ShrayeshCoraça-Huber, Débora C.Patel, VipulĆorić, DankoPater, ZbigniewCorradi, MarcoPathakoti, KavithaCorrea, Cinthia AntunesPatil, SachinCorreia, José A.F.O.Patil, SandeepCorrigan, DamionPato Doldan, BreoganCoskun, BulutPatra, Jayanta KumarCosta Moutinho, FabíolaPatra, NiranjanCosta Rama, EstefaníaPatrick, ErinCosta, Carlos M.Pattanaik, HimansuCostin, GeluPatterson, Albert E.Cottis, BobPatterson, BrianCOURTIAL, Edwin-JofreyPatterson, JenniferCoutinho, Paulo José GomesPattini, FrancescoCovas, JosePauer, WernerCoy, EmersonPauk, JolantaCragg, PeterPaul, AndreaCran, Marlene J.Paul, BiplabCrane, NathanPaul, SanjoyCravanzola, SaraPaul, Suvash ChandraCreatore, MariadrianaPaul, Titan C.Crichton, WilsonPaulino, GlaucioCroenne, CharlesPaulo, AntónioCroissant, JonasPaulsen, Christian OenCrossley, AlisonPaulsen, HaukeCrucho, CarinaPavliček, PavelCsík, AttilaPavlík, ZbyšekCtistis, GeorgiosPavlou, DimitriosCuberes, M. TeresaPavlovic, AnaCuevas, Francisco G.Pawelec, BarbaraCui, FangdaPawlus, PawełCui, HaitaoPečínka, LadislavCui, ZhibinPedersen, HenrikCuisset, ArnaudPedzich, ZbigniewCulliton, DavidPeer, AkshitCycoń, MariuszPegoretti, AlessandroCyganowski, PiotrPei, ZingwayCzaja, P.Peiner, ErwinCzapik, PrzemysławPeiris, T. A. NirmalCzarnecka, BeataPejkowski, ŁukaszCzarnecki, LechPelka, RafałCzarnecki, Slawomir A.Pellegrino, FrancescoCzech, BożenaPellejero, IsmaelCzechowska, JoannaPellicer, EvaCzekanski, AleksanderPellis, AlessandroCzosnek, CezaryPelt, Daniël M.Czujko, TomaszPeña-Méndez, Eladia MD’Addona, Doriana M.Pendergraph, Samuel A.D’Aloia, A.G.Peng, TengD’Anna, FrancescaPeng, WenchaoDa Fonseca, Gláucio SoaresPennelli, GiovanniDa Silva, A. MoreiraPeppel, TimDa Silva, ManelPerale, GiuseppeDa Silva, Pedro RaposeiroPereira Falcon, Juan CarlosDadkhah, MehranPereira Gonçalves, AntónioDaeneke, TorbenPereira, Mauro FernandesDahal, TulashiPereira, Sílvia MoraisDalconi, Maria ChiaraPerepelyuk, MarynaDam, Hieu ChiPERETTI, RomainD’amato, RobertoPérez Del Pino, ÁngelDambone Sessa, SebastianPérez Rodríguez, SaraDamjanović, DarkoPérez Zubiaur, PabloDamma, DevaiahPérez, Esther EnríquezDammaschke, TillPerez-Acebo, HeribertoD’Amora, UgoPérez-Albacete Martínez, CarlosD’Amore, AlbertoPerez-Gavilan, ArianeDa’na, EnshirahPérez-Mendoza, Manuel J.Dana, StancekovaPérez-Moreno, JavierDancette, SylvainPérez-Soriano, Eva M.D’Andrea, AntonioPérigo, Élio AlbertoD’andrea, CristianoPerinelli, Diego RomanoDäne, MarkusPerlepes, Spyros PDanesh-Sani, Seyed AmirPeron, MircoDaneshvar, HosseinPerpiña, XavierDang, AleiPerrella, MicheleD’Aniello, MarioPerrier, JosetteDantan, AurélienPerrin, MarianneDarmanin, ThierryPerrine, Kathryn A.Darvell, BrianPerrot, ArnaudDas, Amit KumarPerteghella, SaraDas, JagotamoyPerumal, SugunaDas, SanjibPerut, FrancescaDas, SujitPessoa, RodrigoDash, BirajaPetcu, CristianDasireddy, Venkata D. B. C.Peter, FranciscDatcheva, MariaPeterson, BrandonDatta, KaustuvPetit, TristanDaugevičius, MykolasPetráková, VladimíraDaus, AlwinPetrella, AndreaDavies, IanPetric, MarkoDavim, J. PauloPetrila, IulianDavis, Daniel C.Petronella, FrancescaDavis, GrahamPetrov, RoumenDavis, PaulPetrov, Yuri V.Davoudinejad, AliPetrů, MichalDavy, JohnPetukhov, Dmitriy I.Dawn, ArnabPeura, PasiDawson, JohnPeyre, PatriceDayen, Jean FrançoisPezzato, LucaDe Aza, Piedad N.Pham, Anh HoangDe Beer, SissiPhan, Hoang-PhuongDe Finis, RosaPhaniraj, M.De Gonzalo, GonzaloPhilipose, UshaDe La Flor, SilviaPhilippe, Régisde Lacalle, Luis Norberto LópezPhoenix, S. LeighDe Maddis, ManuelaPhung, Quoc TriDe Matteis, ValeriaPiana, GiuliaDe Paz, Juan F.Picado-Santos, LuisDe Pertis, MarcoPicard, DonaldDe Riccardis, M. FedericaPiccirillo, ClaraDe Santis, RobertoPiccolo, AntonioDe Silva Indrasekara, AgampodiPickering, EdDe Stefano, LucaPicuno, PietroDebiec, KlaudiaPiechna, JanuszDebliquy, MarcPieczyska, Elzbieta AlicjaDebski, HubertPiedade, Ana PaulaDechow, Paul C.Pierpaoli, MattiaDeferme, WimPieta, PiotrDehghanghadikolaei, AmirPietrikova, AlenaDeimede, ValadoulaPietrusiak, DamianDekel, Dario R.Pietrzak, KornelDel Hoyo, Carmen M.Pietrzyk, BozenaDelaney, ColmPilarczyk, WirginiaDelbe, KarlPilarska, Agnieszka A.Delcamp, JaredPilch-Pitera, BarbaraDelivopoulos, EvangelosPilehvar, ShimaDell’Agli, GianfrancoPilot, RobertoDelle Piane, MassimoPiltan, FarzinDelorme, NicolasPiluso, SusannaDelpouve, NicolasPilyugina, EkaterinaDelrue, StevenPimenov, DanilDelsaute, BricePin, Jean-MathieuDemel, JanPina, João MurtaDemey, HaryPina, SandraDemir, Ali GökhanPindi, SureshDemirci, IbrahimPineda, AntonioDemirocak, Dervis EmrePineda, EloiDemis, SotirisPingitore, NicholasDemoulin, ThibaultPinho, LuísDeng, HonbingPinto, Artur MDennington, SimonPinto, DeesyDenti, LuciaPinto, Deesy GomesDenys, RomanPinzaru, IuliaDeOliveira Vigier, KarinePiotrowski, TomaszDepczynski, WojciechPiquee, JulieDeren, PrzemislawPirani, ChiaraDerrick, MottPirri, AngelaDescartes, SylviePislaru-Danescu, LucianDevaraj, AishwaryaPistone, AlessandroDevasurendra, AmilaPiszczek, PiotrDhakal, AshimPitenis, Angela A.Dhakshinamoorthy, AmarajothiPizzagalli, LaurentDhaliwal, Gurpinder SinghPizzi, AntonioDharavath, SrinivasPizzoferrato, RobertoDhindsa, Kulwinder SinghPlacido, FrankDi Bartolomeo, AntonioPlatt, PhilipDi Battista, DavidePlesca, AdrianDi Bella, GuidoPlott, JeffDi Girolamo, RoccoPlutino, Maria RosariaDi Maio, LucianoPodany, PavelDi Mauro, AlessandroPodila, RamakrishnaDi mundo, RosaPodlaha-Murphy, ElizabethDi Pasqua, Anthony J.Podolsky, JosephDi Sante, RaffaellaPoelman, HildeDi Sarno, LuigiPoeppelmeier, K. R.Di Schino, AndreaPoggio, ClaudioDiana, RositaPokharel, BhadraDiao, ShuoPokharel, PashupatiDias, Rolando CSPolacheck, WilliamDickens, PeterPolanski, MarekDickert, Franz L.Polastri, AndreaDickey, MichaelPolavarapu, LakshminarayanaDieguez Alonso, AlbaPoletto, MatheusDierking, IngoPoliks, MarkDietrich, DagmarPolimeni, AntonellaDiez, Ana GarcíaPolimeni, AntonioDíez-Pascual, Ana MaríaPolowczyk, IzabelaDillert, RalfPółrolniczak, PaulinaDilonardo, ElenaPommer, BernhardDimakis, NicholasPommer, ChristianDimakopoulos, YannisPoncet, SébastienDimos, KonstantinosPons, DirkDing, FeiPons, JosefinaDing, JianxunPontoni, LudovicoDing, LeiPoon, JosephDinia, AzizPop, OanaDinis, Maria Alzira PimentaPopa, LacramioaraDinischiotu, AncaPopa, Mona ElenaDiomede, FrancescaPopat, AmiraliDiraco, GiovanniPopescu, AndreiDiroll, BenjaminPopescu, CrisanDistaso, MonicaPopielarz, RomanDixit, MuditPopovich, V. A.Dixon, DavidPorcarelli, LucaDixon, David A.Poriel, CyrilDjukic, MilosPorojan, LilianaDlouhý, IvoPosadas, PilarDobrikova, AneliaPostigo, Pablo AitorDobroiu, AdrianPostnikov, PavelDobrotă, DanPotgieter, HermanDobrovodský, JozefPoulikakos, LDDobslaw, DanielPoulios, Ioannis G.Dogossy, GáborPoupin, ChristopheDolata, Anna JaninaPouponneau, PierreDoležal, PavelPourrahimi, Amir MasoudDolocan, AndreiPoveda Bautista, ElisaDománková, MáriaPovoden-Karadeniz, ErwinDombek, GrzegorzPoyato, RosalíaDomenici, ValentinaPrabhu, VivekDomine, MarceloPradeep, Konda GokuldossDomínguez-Leal, María IsabelPradhan, Dhiren K.Donchev, AlexanderPrado-Gonjal, JesúsDong, H BPrajer, MarekDong, MingdongPrakash, AdithyaDong, ZhizhongPrakash, ArunDorfs, DirkPramanik, BrahmanandaDorin, ThomasPramanik, SandipanDorman, JamesPramudita, Jonas A.Dorner-Reisel, AnnettPrasad, BhagwatiDoroudiani, SaeedPrasath, V. B. SuryaDorozhkin, Sergey V.Prashanth, Konda GokuldossDorval Courchesne, Noémie-ManuellePrates, PedroDos Santos, Rui GalhanoPrati, SilviaDos Santos-García, A. J.Praticò, Filippo GianmariaDosbaeva, G.K.Prida, Víctor ManuelDou, JinboPriecel, PeterDow, Hwan SooPriel, EladDraganovska, DagmarPrieto, CarlosDraheim, Roger R.Prince, Roger C.Drevet, RichardPrisco, UmbertoDrewniak, SabinaPrlić Kardum, JasnaDrobná, HelenaProcek, MarcinDrozdenko, DariaProch, SebastianDrummer, DietmarProcházka, MarekDruzbicki, KacperProcházková, L.Du Roscoat, Sabine Rolland OllandProchowicz, DanielDu, FanfanProvis, JohnDu, Jia-ChongPruna, AlinaDuan, LuchunPruncu, Catalin IulianDuarte Campos, Daniela FilipaPrusov, EvgenyDubenko, IgorPryamikov, Andrey D.Dubey, AshishPryamitsyn, Victor A.Dubey, RichaPrzestacki, DamianDubey, ShriPrzybylek, PiotrDuchesne, MarcPshyk, AleksandrDuchosal, ArnaudPsomopoulos, Constantinos S.Dudin, PavelPsyllaki, PandoraDudina, DinaPszczola, MarekDudina, Dina V.Ptak, MariuszDugmore, ThomasPu, Nen-WenDuisit, JeromePu, Ying-ChihDumas, JeraldPucci, AndreaDumée, LudovicPuchades, IvanDumitran, Laurenţiu MariusPuchalski, MichalDumitrica, TraianPuga, Alberto V.Dumitriu, CristinaPuga, HélderDumont, Pierre J.J.Puglia, DeboraDunderdale, Gary J.Pui, AurelDunne, NicholasPujadas, PabloDuong, LinhPuli, VSDuprez, DanielPulido, DavidDurach, MaximPullano, Salvatore AndreaDuran-Valle, Carlos JavierPunzo, FrancescoDurejko, TomaszPurcell-Milton, FinnDurose, KenPurwasih, NiningD’Urso, GianlucaPustan, MariusDuske, KathrinPuttreddy, RakeshDušková-Smrčková, MiroslavaPyo, SukhoonDutta, AbhishekPyrgaki, KonstantinaDutta, AniruddhaQi, QunfenDutta, BiswanathQi, YuboDutta, PalashQiang, ZheDuy, Le ThaiQin, HantangDyatkin, BorisQuero, GiuseppeDyck, Ondrej E.Quijano, LauraDymek, StanisławQuintana, RobertDyzia, MaciejQuintero, FélixDzakpasu, MawuliQutachi, OmarDziewit, LukaszKong, QingzhaoDziki, DariuszR N Correa, DiegoDzimitrowicz, AnnaRaabe, DierkDzioba, IhorRabczuk, TimonDzionk, StefanRabe, Karin M.Dziuba, DmytroRäbiger, DirkDziubla, ThomasRabnawaz, MuhammadDzolic, ZoranRaccichini, RinaldoEbara, MitsuhiroRace, MarcoEblagon, KatarzynaRackauskas, SimasEbrahimi, AidaRaczkiewicz, WiolettaEbralidze, Iraklii I.Radek, NorbertEbramzadeh, EddieRadonic, VasaEciolaza, LukaRadtke, AleksandraEcker, WernerRadu, DorinEddy, LiyantoRǎducanu, DoinaEdgar, James H.Raeis Hosseini, NiloufarEdström, DanielRafsanjani, AhmadEduok, UbongRagab, KhaledEgan, Paul F.Ragab, TarekEgberts, PhilipRagusa, AndreaEggenhöffner, RobertoRahier, HubertEgodawatte, ShaniRahimi, FaridEgorkin, Vladimir S.Rahman Rashid, Rizwan AbdulEhrmann, AndreaRAHMAN, Md MostaqurEhtemam Haghighi, ShimaRahman, Mohammad ShafiqurEhtemam-Haghighi, ShimaRahman, Seikh M.H.Eickenscheidt, MaxRahme, KamilEinarsson, ErikRahnama, AlirezaEisenbart, MiriamRai, AmriteshEjfler, JolantaRai, PrakashEkielski, AdamRaimondo, M.Ekinci, HuseyinRaj, Pulugurtha MarkondeyaEklund, PerRajala, PauliinaEkpeni, LeonardRajapandiyan, PanneerselvamEl Badawy, AmroRajaraman, SivaramakrishnanEl Moumen, A.Rajaraman, SwaminathanElahi, HassanRajca, MariolaElahinia, MohammadRaju, KatiElaissari, AbdelhamidRakhmonov, JovidEl-Gendy, Ahmed A.Ramadoss, AnanthakumarElhag, SamiRamakrishnan, SathishEl-Hofy, MohamedRamasamy, MohankandhasamyElias, AlexRamire-Castellanos, JulioElias, Ana LauraRamirez Miquet, EvelioElias, JanRamis, GianguidoEliyan, FaysalRamos, DanielEliyan, Faysal FayezRamos, PatriciaElkasabi, YaseenRamos-Ortiz, GabrielEl-Kazzi, SalimRampazzo, ElenaEllinas, KosmasRana, Abu Ul Hassan SarwarElliott, Amelia M.Rana, DipakElliott, BrentRanguelov, BogdanElmi, ChiaraRaniszewski, GrzegorzEl-Safty, Sherif A.Ranjit, SumanElsharafi, MahmoudRanjitkar, SarbinEmberley, RichardRanjkesh, AmidEnachescu, CristianRantuch, PeterEndruweit, AndreasRao, AgnėEngel, DieterRao, AshitEngelke, HannaRao, SmithaErba, AlessandroRapiejko, CezaryErbas, AykutRaposo, MariaErcegović Ražić, SanjaRashidi, ArmanErdem, EmreRastogi, Vibhore KumarErdeniz, DincRathod, VivekEremin, ArtemRatnakumar, B. V.Erhart, JiriRatova, MarinaErna, DelczegRau, Julietta V.Erts, DonatsRaucci, Maria GraziaEscorihuela, JorgeRaudino, AntonioEscudero, CarlosRaut, SangramEsen, CemalRautkari, LauriEser, SemihRavaioli, StefanoEsin, Vladimir A.Razavi, PedramEslami, HosseinRazavi, Seyed Mohammad JavadEslamian, MortezaReber, ChristianEspinach, Francesc XavierRecek, NinaEsposito Corcione, CarolaRecord, Marie-ChristineEsposito, ElisaReddy, AmaranathaEsposito, LucaReddy, Govardhan N.ManjunathaEsteban-Fernandez De Avila, BertaRefaely-Abramson, SivanEstelle, PatriceRege, KristenEsteves, Bruno MReguero, MarEstokova, AdrianaRehnlund, DavidEstrany, FrancescReid, MarkEtxabide, AlaitzReinke, Gerald H.Evtugyn, GennadyReinoso, JoseFabbrocino, FrancescoReis, P. N. B.Faber, CarinaReiser, OliverFabiano, EduardoReisman, Scott A.Fabisiak, KaziemierzRemediakis, IoannisFabre, BrunoRen, DingkunFacchini, FrancescoRenault, Pierre-OlivierFadhel, MustafaRenk, OliverFajín, José L. C.Rennhofer, HaraldFalat, LadislavRenterghem, Wouter VanFalco, AlbertoReshetenko, Tatyana V.Faleschini, FloraRethmeier, MichaelFambri, LucaRevenant, ChristineFan, BinRevuru, Rukmini SrikantFan, Pui L.Rew, YounhoFancher, Chris M.Rhee, InkyuFang, Li-WenRho, JunsukFang, Ronnie H.Rhyee, Jong-sooFanteria, DanieleRibeiro Pereira, Luiz FernandoFantoni, FrancescaRibeiro, Ana Paula C.Fantozzi, GilbertRibeiro, JoãoFantuzzi, NicholasRibeiro, Maria HFara, LaurentiuRiccardi, ChiaraFaraldos, MarisolRicciardella, FilibertoFare, SilviaRicciotti, LauraFargnoli, MarioRichaud, EmmanuelFaria Albanese, Jimmy AlexanderRider, A.N.Farquhar, AnnaRidi, FrancescaFarrokhpanah, AmirsamanRiess, GisbertFatyeyeva, KaterynaRighi, Maria CleliaFaulkner, R. G.Rigo, EvelineFauzi, IlianaRiha, ShannonFayaz, MohammadrezaRiha, Shannon C.Faye, OmarRim, You SeungFazeli, FatehRimoldi, LucaFechner, MichaelRimondini, LiaFedorko, GabrielRinnan, ÅsmundFeito, NorbertoRíos, JoséFekete, ZoltánRipamonti, DarioFeld, ArturRitmi, SamiFeldshtein, Eugene ÉRitz, UlrikeFelgueiras, HelenaRiu, Doh-HyungFeliu, SebastianRives, VicenteFellows, ChrisRivière-Lorphèvre, EdouardFeng, CaiRizal, ConradFeng, ChuangRizzi, Gian AndreaFeng, Sheng-WeiRizzolio, FlavioFeng, TianliRoa Rovira, Joan JosepFeng, XundaRobbennolt, ShaunaFernandes, Maria HelenaRobert, EricFernández Vidal, Severo RaúlRoberto, DominiqueFernández, Belén DíazRoberts, NicholasFernandez, José JesúsRocha, Fernando T.F.T.Fernandez-Garcia, JavierRoche, Jean-MichelFernández-García, MartaRockstroh, NilsFernandez-Garcia, RaulRodenbough, PhilipFernandez-Morales, Francisco JesusRodger, James A.Feron, KrishnaRodiouchkina, MariaFerone, ClaudioRodrigues, CelestinoFerrandiz Bou, SantiagoRodrigues, Celia F.Ferrara, ChiaraRodrigues, FernandaFerrara, LiberatoRodriguez Couto, SusanaFerrari, MicheleRodríguez Millán, MarcosFerreira, AiresRodriguez, Antonio RiveiroFerreira, Ana MarinaRodriguez, NoelFerreira, FabioRodríguez-Castellón, EnriqueFerreira, Manuel MarquesRodriguez-Ibabe, Jose MariaFerreira, Marta S.Rodríguez-Lozano, Francisco JavierFerrucci, MassimilianoRodríguez-Mirasol, JoséFerry, CécileRodziewicz, JoannaFettkenhauer, ChristianRoggendorf, HansFiedler, BodoRogl, GerdaFigueira, RitaRogl, Peter FranzFigueira, Rita BacelarRogovoy, Anatoly A.Figueiral, Maria HelenaRoh, ChanghyunFihey, Jean-LucRoh, Jong-wookFijałkowski, KarolRohnke, MarcusFilip, PetrRokosz, KrzysztofFillipov, SergeyRokstad, Anne MariFini, StefanoRomán-Martínez, M.C.Finney, Eric E.Romano-Chernac, Fabian B.Finocchio, ElisabettaRomanowicz, PawelFinšgar, MatjažRomar, HenrikFiorati, AndreaRomero, Aldo HumbertoFiorenza, RobertoRomero, PedroFirestein, KonstantinRomero-Muñiz, CarlosFirlak, MelikeRomero-Salguero, Francisco J.Firrincieli, AndreaRonca, AlfredoFischer, Christian B.Roozbahani, MahdiFischer, Hartmut R.Ropital, FrançoisFischer, HolgerRoppolo, IgnazioFischer, PeterRosa, Angela Daniela LaFischer, Reinhard XRosas, OmarFlandin, LionelRosenzweig, DerekFlege, StefanRoseti, LiviaFleury, EricRossella, FrancescoFlorczyk, Stephen J.Rossi, Cesare OlivieroFlores-Colen, InêsRossignol, JulienFlox, CristinaRosso, MarioFoa Torres, Luis E. F.Rosu, Marcela-CorinaFodor, CsabaRoth, JohannesFonollosa, JordiRöttger, ArneFonseca, AnaRousakis, TheodorosFontanari, VigilioRowthu, SriharithaForaboschi, PaoloRoy, AnupamForcada, LaiaRoy, SagarForget, AurelienRoy, TaniaFormela, KrzysztofRoy, Utpal NFornell, JordinaRóżalska, BarbaraForner, LeopoldoRozga, PawelFörster, WolfgangRozman, NejcFörsth, MichaelRubini, SilviaForsthuber, BorisRubino, GuidoFortea-Verdejo, MartaRubio, Ramón G.Foucher, DanielRucka, MagdalenaFousova, MichaelaRudawska, AnnaFox, StephenRudolf, RebekaFraga, Mariana AmorimRudykh, StephanFragassa, CristianoRuffino, FrancescoFraile, AlbertoRuggieri, FabrizioFrancesco, NegroRughoobur, GirishFrancesconi, LucaRuiz Rubio, LeireFrancia, CarlottaRuiz, M. PilarFranco, LourdesRumyantseva, Marina N.Frantziskonis, George N.Rung, StefanFrazier, CharlesRunge, Jude MaryFrazzica, AndreaRusponi, StefanoFreakley, Simon J.Russell, Alan M.Freudenberger, JensRusso, PietroFriák, MartinRusso, SalvatoreFriedrich, FrankRuths, MarinaFriedrich, SemiramisRuys, AndrewFrignani, AlessandroRyadnov, MaxFrisenda, RiccardoRyan, James G.Fritzsche, SiegfriedRyan, Michael P.Fröhlich, ThomasRybakov, Kirill I.Froitzheim, JanRybiński, PrzemysławFrontera, AntonioRydosz, ArturFrontera, PatriziaRyo, InoueFu, TengfeiRyou, JaesukFu, ZhezhenRytöluoto, IlkkaFuchs-Godec, ReginaSaafi, MohamedFuente, Germán F. De LaSaalwachter, KayFujii, YoshihisaSABBAN, ALBERTFujita, SatoshiSaboori, AbdollahFukuda, TakeshiSabri, YliasFukushima, JunSacco, AdrianoFulvio, Pasquale F.Sacco, OlgaFulvio, Pasquale FernandoSacher, EdwardFuoco, AlessioSadeghi, EsmaeilFuoco, TizianaSadeghifar, HamidrezaFuso, FrancescoSadowska, BeataFydrych, DariuszSadowski, ŁukaszFytas, GeorgeSaedi, SoheilGaan, SabyasachiSaeidi, KamranGabay, AleksandrSaffari Pour, MohsenGabay, Alexander M.Safiuddin, Md.Gabitov, RinatSah, Santosh PrasadGac, WojciechSaha, BiswajitGadisa, Abay DinkuSaha, GobindaGafurov, MaratSaha, ShyamaliGagarin, AlexanderSaharan, LalitaGagliardi, FrancescoSahu, DebashishGain, Asit KumarSai, HiroakiGajjar, ChiragSaid Schicchi, DiegoGala, Rikhav PSaielli, GiacomoGalanakis, IosifSain, SunandaGalatus, RamonaSainsbury, TobyGaldiero, EmiliaSaito, KazuyaGalenda, AlessandroSaito, NoritakaGalenko, PeterSaito, TakeshiGalgalikar, RohanSaitoh, AkiraGalizia, PietroSaiz, FernanGalkin, MaximSajjadian, MasoudGalkin, Nikolay GennadevichSakagami, KimihiroGallego, AntolinoSakai, JoeGallego, JuanSakai, ToshioGallei, MarkusSakharova, Nataliyagallo, pasqualeSakidja, RidwanGalvão, IvanSakon, TakuoGama Goicochea, ArmandoSałaciński, TedeuszGambarova, Pietro G.Salahub, DennisGan, Yong X.Salamanca, Constain H.Gancarczyk, AnnaSalameh, ChrystelleGanczarski, Artur WładysławSALAPARE, Hernando IIIGanewatta, MitraSalas, CarlosGangishetty, MaheshSałasińska, KamilaGangloff, SophieSalehi, SaeedGanta, DeepakSalerno, AurelioGantenbein, BenjaminSalerno, MarcoGao, PeiyuanSalguero, JorgeGao, TongchuanSaligram, Shreyasgao, xiaojianSalili, Seyyed MuhammadGao, ZheSalimi Jazi, MehdiGarbiec, DariuszSalmeia, Khalifah A.Garbin, EnricoSalmi, MikaGarboczi, Edward J.Saltas, VassilisGarbovskiy, YuriySalvador, JamesGarbrecht, MagnusSalvalaglio, MarcoGarcía Calvo, José LuisSalvati, EnricoGarcía Martínez, TomasSalzano De Luna, MartinaGarcia, C. IssacSamanidou, Victoria FGARCÍA, DANIELSamanta, SubarnaGarcía, NuriaSamaras, IoannisGarcia, RosarioSamborski, SylwesterGarcia-Escorial, AsuncionSammelselg, VäinoGarcía-González, Carlos A.Samoila, P. MugurelGarcía-Martínez, Jesús-MaríaSamuel, Fawzy-HosnyGarcía-Moreno, OlgaSamuele, LilliuGarcia-Pardo, JavierSamuha, ShmuelGarcia-Tecedor, MiguelSamulionis, VytautasGardy, JabbarSan Fabian, EmilioGarg, NishantSanati, MahdiGariboldi, ElisabettaSánchez, AlfredoGaroufalis, ChristosSanchez, AntonioGaska, KarolinaSánchez, AntonioGaudiuso, RosalbaSánchez, IsidroGaudry, ÉmilieSánchez-García, DavidGavioli, LucaSánchez-Somolinos, CarlosGavira, JoséSánchez-Soto, Luis L.Gavras, SergeSanchís, María JesúsGawlik, Marcjanna MariaSancho, AnaGazo, RadoSandri, GiuseppinaGeatches, DawnSangiorgi, CesareGeijselaers, Hubert J.M.Sanin, Vladimir N.Geisler-Lee, JaneSanjuán, Miguel ÁngelGelbstein, YanivSankaran, Kamatchi JothiramalingamGenchi, GiadaSankarasubramanian, ShrihariGeng, LinxiaoSantamaria, MonicaGenova, TullioSantecchia, EleonoraGenovese, DamianoSanthiyagu, Vijay JosephGentile, PiergiorgioSantodonato, LouGenty, EricSantoni, FabioGenuino, Homer C.Santoni, IlariaGeorgantzinos, S.k.Santos, AbelGeorge, Thomas F.Santos, João M.Georgieva, RadostinaSantos, Luís PicadoGeorgiou, EmmanuelSantos, Maria JacintaGeorgiou, Emmanuel P.Santos-Carballal, DavidGeorgopanos, ProkopiosSantulli, CarloGerman, Konstantin E.San-Valero, PauGervais, FrançoisSanz Garcia, AndresGettleman, LawrenceSanz Pont, DanielGhabchi, RouzbehSanz, Alicia ManjonGhaeli, ImaSanz, EloyGhahremaninezhad, AliSaoud, KhaledGhandehariun, A.Sapudom, JiranuwatGhanmi, HelmiSarac, BaranGharbi, Mohamed AmineSarasini, FabrizioGhatak, KamalikaSaremi Yarahmadi, SinaGhazi-Nezami, FarnazSaremi, MehdiGherardi, FrancescaSarkar, MohanGheribi, Aimen.E.Sarkar, SujanGherlone, EnricoSarker, DyutiGhidelli, MatteoSarlin, EssiGhobadi, EhsanSarma, SauravGhods, MasoudSarswat, Prashant K.Gholamalizadeh, EhsanSartori, NeimarGhosh, AnindyaSasai, YasushiGhosh, MonojSasaki, Jun-IchiGhosh, SohamSato, HisashiGhoshal, SushantaSato, KimiyasuGhyngazov, Sergey AnatolievichSato, TakayaGiacomazza, D.Sato, TakuichiGiannakas, ArisSavaniu, CristianGiannakoudakis, DimitriosŠavija, BrankoGiannazzo, FilippoSawabe, KyoichiGiannella, VenanzioSawada, HideakiGiasin, KhaledSaxena, SaurabhGiaume, DomitilleScalet, GiuliaGiberti, HermesScarano, AntonioGibson, ChristopherScarfato, PaolaGibson, IanScarponi, ClaudioGierszewska, MagdalenaScendo, MieczyslawGietzelt, ThomasŠčetar, MarioGigot, ArnaudSchabowicz, KrzysztofGil, BarbaraSchaffoener, StefanGill, Richie H.S.Schartel, BernhardGillan, Edward G.Scheeline, AlexanderGillich, Gilbert-RainerScheffler, MichaelGiner-Casares, Juan J.Scheinherrová, LenkaGiorgio, IvanSchenk, ThomasGiri, ParitoshSchiavon, MarcoGirolami, MarcoSchiavone, GiuseppeGiubileo, FilippoSchierbaum, Klaus DieterGiunta, GaetanoSchiraldi, DavidGivehchi, RahelehSchiraldi, David A.Gizdavic-Nikolaidis, MarijaSchirhagl, RomanaGkanas, EvangelosSchirp, ClaudiaGlaeser, JessieSchledjewski, R.Glinicki, Michał ASchmeer, SebastianGloc, MichalSchmidlin, PatrickGloria, AntonioSchmidt, BernhardGłowacki, MichałSchmidt, JochenGłuchowski, AndrzejSchmitt, KatrinGlüsen, AndreasSchneider, ChristofGnade, Bruce E.Schneider, MichaelGniewosz, MałgorzataSchneider, RaphaelGnyba, MarcinSchneider, RaphaëlGockenbach, ErnstSchneller, TheodorGodeau, GuilhemSchoedel, AlexanderGodwin, IanSchönemann, LarsGoel, SarikaSchöppner, VolkerGoenezen, SevanSchowalter, MarcoGoff, H. DouglasSchrade, MatthiasGoh, Kheng-LimSchrittwieser, StefanGohs, UweSchuetz, MartinGok, OzgulSchuh, ChristinaGola, ArkadiuszSchulz, BurkhardGolaszewski, JacekSchulz, FlorianGoldberg, Margarita A.Schwaminger, SebastianGoldoni, MatteoSchwan, MarinaGolewski, Grzegorz LudwikSchwan, WilliamGolgovici, FlorentinaSchwarz, KarlheinzGolombek, KlaudiuszSchweiss, RuedigerGolovin, Igor S.Schwentenwein, MartinGoltz, Douglas M.Sciascia, LucianaGolub, IgorScirè, SalvatoreGombac, ValentinaScoccia, RossanoGomes, Ana P.Scotognella, FrancescoGómez De Salazar, José MaríaScotti, AmericoGómez-Graña, SergioScribano, VittorioGómez-Ruiz, SantiagoScribante, AndreaGómez-Soberón, José ManuelScrimin, PaoloGómez-Tejedor, José AntonioScutelnicu, ElenaGonçalves, AnaSealy, MichaelGonçalves, Lidia M DSecu, Elisabeta-CorinaGondolini, AngelaSecu, MihailGong, HaijunSedelnikova, Mariya B.Gong, PanSedev, RossenGong, YongkangSedlacek, MarkoGonilha, José ASedlačík, MichalGonome, HirokiSedlák, PetrGonzález Briones, AlfonsoSeetharaman, SankaranarayananGonzález Morán, Carlos OmarSegal, Vladimir M.González Pérez, IsaíasSegreto, TizianaGonzález, IsraelSeguí, ConcepcióGonzález, SergioSeidel, Rüdiger W.Gonzalez-Gutierrez, JoaminSeiersten, MarionGonzález-Sánchez, M. I.Seifan, MostafaGonzalo Orden, HernánSeifitokaldani, AliGoo, Byeong-ChoonSeiner, HanušGopalan, Sai AnandSeino, SatoshiGoraus, JerzySeipenbusch, MartinGordiichuk, PavloSeitl, StanislavGoreham, ReneeSekiguchi, YuGórny, MarcinSelf, EthanGorseta, KristinaSellami, NazmiGostomski, PeterSellier, Jean MichelGoswami, NirmalSelyanchyn, RomanGoto, YosukeSemaltianos, Nikolaos G.Gotor Fernandez, VicenteSemenova, Irina P.Goutam, ShovonSemjonov, SergeyGraaf, H Van DerSenatov, Fedor S.Grabowski, MarcinSenderowski, CezaryGraciani, EnriqueSenos, Ana Maria De Oliveira RochaGraczykowski, BartlomiejSeo, JihoonGragnani, Gian LuigiSeo, YongbeomGrajcar, AdamSeok, HyunGrajek, HenrykSepe, RaffaeleGrama, SilviaSepulveda, AbdonGrande, CarlosSequeira, CésarGrandjean, AgnèsSerban, NicolaeGranet, RobertSergey, MakarovGranitzer, PetraSergi, VincenzoGrasso, SalvatoreSerikov, A.Grau, GerdSerizawa, AiGraves, JosephSerrano, Dolores R.Graziani, AndreaSesana, RaffaellaGraziani, GabrielaSestoke, JustinaGreen, DavidSeung Tae, ChoiGreer, Andrew I. M.Seung, Yoon RyuGregor-Svetec, DianaSeyed Amid, TahamiGreiner, ChristianSeyyed Monfared Zanjani, JamalGribniak, ViktorSgambitterra, EmanueleGric, TatjanaSgroi, Mauro FrancescoGriffiths, MalcolmShah, AashishGrigsby, Christopher L.Shah, SandeepGrivel, Jean-ClaudeShaha, SugribGrizzuti, NinoShaha, Sugrib KumarGroch, PawełShahani, AshwinGrossi, MarcoShahmoradi, MahdiGrugeon, SylvieShahnewaz, MdGrumezescu, Alexandru MihaiShahpari, MortezaGrutter, AlexanderShalaby, MohamedGrützmacher, Philipp G.Shams, AlirezaGrydin, OlexandrShamsaei, EzzatollahGrzegorz, KokotShan, SicongGrzybowski, AndrzejShanjani, YaserGuariglia, EmanuelShankar, EswarGüemes, AlfredoShanks, Robert AGueorguiev, G.K.Shao, ChenhuiGueorguiev, Gueorgui K.Shapter, JosephGuerra, GaetanoSharkeev, YuriiGuessasma, SofianeSharma, BhupendraGuex, Anne GéraldineSharma, DeeptiGui, MinghuiSharma, ManishaGuibal, EricSharma, PankajGuillou, FrancoisSharma, SwatiGuiot, CaterinaSharma, YogeshGulaim A., SeisenbaevaShaygan, MehrdadGulari, ErdoganShehata, NaderGulgunje, PrabhakarSheikh, SaadGunawidjaja, RayShemesh, MosheGun’ko, Vladimir MShen, Kelvin K.Guo, ShuangzhuangShen, Yun-HweiGuo, WeiSher, PraveenGuo, YuanhaoSherbakov, SergeiGuo, ZhanhuSheri, MadhuGupta, AniruddhaShevtsov, SergeyGupta, AnkurShi, JieGupta, KajalShi, SuanGupta, MohitShi, XianmingGupta, PrachiShi, XiaowenGupta, Ram K.Shi, XijunGupta, SanjuShibkov, A. A.Gurzhiy, Vladislav V.Shie, Ming-YouGuselnikova, Olga AndreevnaShields, PhilipGutekunst, Will R.Shigeishi, MitsuhiroGutruf, PhilippShilko, Evgeny ViktorovichGuzanová, AnnaShilo, DoronGuzman, MarceloShim, JiwookGwalani, BharatShimada, KeitaHa, Heon-YoungShimizu, MakotoHabisch, StefanShimoi, NorihiroHabrat, WitoldShin, DongwookHadavinia, HomayounShin, KwanwooHadermann, JokeShin, Weon HoHadjiargyrou, MichaelShin, YooseokHaffner-Luntzer, MelanieShinoda, KentaroHafuka, AkiraShinya, NorioHagenmuller, PascalShiogai, JunichiHaghdadi, NimaShirokov, Vladimir B.Hahn, Bernadette N.Shirvanimoghaddam, KamyarHahn, Jae RyangShishkin, AndreiHaidemenopoulos, GregoryShishkovsky, IgorHaider, SyedShiue, Ren-KaeHaitjema, HanShoffstall, AndrewHajilar, ShahinShokouhimehr, MohammadrezaHajžman, MichalShokrani, AlborzHakimi, OrlyShrestha, Lok KumarHakkarainen, MinnaShroff, PiyushHalada, GaryShtepliuk, IvanHalder, AvikShterenlikht, AntonHall, ColinShu, ShipengHall, SteveShunmugasamy, Vasanth C.Hallett-Tapley, GenieceShvartsman, VladimirHallstedt, BengtSicakova, AlenaHallsworth, J.E.Sidorenko, AlexanderHalmešová, KristýnaSidorenko, Daria A.Hamad, KotibaSiedlecka, EwaHamann, EliasSierra-Fernandez, A.Hamdana, GerrySievenpiper, DanHamidi, Youssef K.Signore, GiovanniHamila, NahieneSikirzhytski, VitaliHamilton, B. CarterSikora, PawelHamilton, CarterSikorska, WandaHammersley, SimonSikorski, WojciechHamon, Jean-RenéSilikas, NickHamon, MorganSiller, Hector R.Hamuyuni, JosephSilva, Francisco J. G.Han, DingSilva, HugoHan, Dong-WookSilva, Rui VascoHan, Min-CheolSilva, Teresa Moura EHanaor, DorianSilvestroni, LauraHanaor, Dorian A HSima, LiviaHanawa, TakaoSimani, SilvioHandzlik, PiotrSimha Martynkova, GrazynaHanks, TimothySimion, Cristian E.Hänninen, HannuSimka, WojciechHansen, ChristopherSimm, Thomas HadfieldHansen, Klaus SchüttSimmonds, Paul J.Hao, YongSimocko, ChesterHaouas, MohamedSimoes, MarioHaque, RezwanulSimon, ChristianHarada, ShuntaSimonetti, GiovannaHarada, YukihiroSims, HunterHarding, IanSimserides, ConstantinosHardy, John G.Šimůnek, AntonínHare, ColinSimunovic, KaticaHarhash, MohamedSinatra, Nina RoseHarijan, Rajesh KSinebryukhov, Sergey LeonidovichHariri-Ardebili, Mohammad AminSing, Swee LeongHarja, MariaSingh, AlokHarkness, PatrickSingh, AshishHarničárová, MartaSingh, D.P.Haro, MartaSingh, GurpreetHarris, PeterSingh, HarpalHärtel, MarkusSingh, Jitendra KumarHärtel, SebastianSingh, PrashantHasanian, MostafaSingh, RobinHasiuk, FranciszekSingh, SubhashHassan, FirasSingh, YogeshHassan, Hamad UlSingla, MithunHassan, Sammer-ulSinha, JasmineHassanin, HanySioutopoulos, DimitriosHassel, ThomasSiracusa, ValentinaHata, TamakoSirvio, JuhoHatada, RurikoSiska, FilipHattar, KhalidSittner, PetrHattori, TomokazuSiwak, PiotrHaurie Ibarra, LaiaSiwińska-Ciesielczyk, KatarzynaHausmann, JoachimSkaltas, TheodosisHawk, J. A.Skapin, Sreco D.Hayakawa, TohruSkorik, Yury A.Hayase, MasanoriSladek, VladimirHayashi, FumitakaSlesarenko, ViacheslavHayashi, HisashiSlifka, Andrew J.Hayashi, KeiSloma, MarcinHayashi, ShigenariSlosarczyk, AgnieszkaHayashi, YoshihitoSmaga, MarekHaynes, DavidSmani, YounesHe, WenhanSmarzewski, PiotrHector, Andrewsmet, philippeHedayati, AliSmetana, VolodymyrHee, Ay ChingSmiatek, JensHegab, HussienSmith, Michael A.Hegedűs, CsabaSmith, TimothyHeimann, RobertSmolina, IrinaHein, JoachimSmolyaninov, IgorHeinzel, CarstenSmulders, MaartenHeise, RainerSobaszek, MichałHeitkam, SaschaSobiesiak, MagdalenaHejna, AleksanderSobolev, KonstantinHelmer, Harald ErnstSobus, JanHelsch, GundulaSödergård, AndersHelseth, LarsSoejima, TetsuroHemraz, Usha D.Sohn, Seok SuHenderson, Luke C.Sohrabpoor, HamedHennessy, JohnSoin, NavneetHeredia-Guerrero, José AlejandroSokolova, Maria P.Hermawan, HendraSola, RamonaHermes, MichielSolano, FranciscoHermoso-Orzáez, Manuel JesúsSolioz, MarcHernández Saz, JesúsSolitro, GiovanniHernández, Nacú B.Solitro, Giovanni FrancescoHernandez-Vazquez, Jesus-MariaSoman, RuturajHernik, BartłomiejSomayazulu, MadduryHerrmann, HeikoSomireddy, MadhukarHerrnsdorf, JohannesSong, ChangsikHerzog, Joseph B.Song, ChengHesebeck, OlafSong, Guang-LingHidalgo, JavierSong, HyunwookHidalgo-Manrique, PalomaSong, KenanHildreth, BlakeSong, MiaoHill, DanielSong, XuanHilloulin, BenoitSónia, SimõesHills, RonaldSonnier, RodolpheHilšer, OndřejSontakke, Atul DHilton, RamseySorrell, J. MichaelHinderliter, BrianSorrentino, AndreaHindra, HindraSorrentino, LucaHintennach, AndreasSorrentino, LuigiHintzen, HubertusSotelo-Vazquez, CarlosHippler, RainerSotgiu, GiovanniHirai, TomoyasuSotiropoulos, SotirisHirano, HidekiSoto, RobertHirata-Tsuchiya, ShizuSotome, MasatoHirayama, FumitoshiSotta, PaulHirosawa, ShoichiSougrati, Moulay TaharHirota, YuichiroSoukal, FrantisekHirsch, ThomasSoundappan, ThiagarajanHirtz, MichaelSounthararajah, ArooranHlobil, MichalSouravlas, StavrosHmaidouch, RimSovják, RadoslavHnida, KatarzynaSpanhel, LubomirHo, Wen-JengSpeicher, MagdalenaHobbs, Michael L.Spendier, KathrinHobosyan, MkhitarSperanzini, EmanuelaHoch, ConstantinSperling, Laura ElenaHoetzer, JohannesSpiess, LotharHoffman, JasonSpigarelli, StefanoHoffmann, AndreasSpina, RobertoHofmann, KathrinSpinazze, AndreaHojná, AnnaSpinella, NinoHokamoto, KazuyukiSpišák, EmilHolewinski, AdamSpoerk, MartinHoll, HelmutSportelli, Maria ChiaraHoller, StephenSqueglia, NunzianteHolynska, MalgorzataSrinivasan, GowrishankarHomaeigohar, ShahinSrivastava, G.pHomola, TomášSrivatsa, Srikanth ChakravartulaHong, Chang-HeeSroka, MarekHong, Ki-namStaab, Torsten E. M.Hong, MinStachiv, IvoHong, Suck WonStachurski, Z.H.Hong, SukjoonStacul, StefanoHong, Sung HwanStagon, Stephen PeterHong, Won SikStahl, UllrichHonus, StanislavStaicu, TeodoraHorak, EmaStan, GEORGEHorrocks, A. RichardStan, Miruna SilviaHorsley, SimonStanca, Sarmiza ElenaHorszczaruk, ElżbietaStanciu, Mariana DomnicaHorvat, JosipStancu, CristinaHorwat, DavidStanek, AgataHosein, YaraStanescu, DanaHosier, Ian LStankevic, VoitechHoskins, ClareStarostenkov, MikhailHosohata, KeikoStassen, IvoHošovský, AlexanderStavropoulos, PanagiotisHospodková, AliceStavroulakis, Georgios E.Hossain, EklassStawarz, MarcinHossain, KamalStawska, HannaHosseini Nejad, ElhamSteensels, RikHosseinnezhad, ShahrzadSTEFANOIU, RaduHosseinpour, SamanSteinberg, SimonHosseinpourpia, RezaStepanov, Gennady V.Hostaš, JiříStepanov, Nikita D.Housecroft, CatherineStephan, DietmarHouska, JiriStephan, HolgerHowell, BobStephenson, David A.Hoyos, PilarStetter, RalfHrnjak-Murgić, ZlataSteudel, SoerenHryniewicz, TadeuszStewart, David M.Hsiao, Chun-ChingStine, Keith J.Hsieh, Chih-ChunStjepanović, MarijaHsieh, Raymond J.Stobiecka, MagdalenaHsieh, Wen-ChuanStock, StuartHsu, Cheng-HsunStöckelhuber, Klaus WernerHsu, Fu-YinStoleru, ElenaHsu, Kuo-ShengStolov, Andrei A.Hsu, Quang-CherngStolyarov, Vladimir VladimirovichHsu, Yu-KueiStommel, MarkusHsueh, Yi-HuangStopic, SreckoHu, HelenStorti, EnricoHu, Hsin-HuiStorti, GiuseppeHu, KesongStoychev, GeorgiHu, NingStraka, L’uboslavHu, YangStrankowski, MichałHuang, Chih-ChiaStraumal, BorisHuang, Chi-YenStraumal, Boris B.Huang, CuiStrek, TomaszHuang, Genin GaryStrelniker, Yakov M.Huang, JianweiStreubel, RobertHuang, JianyingStrini, AlbertoHuang, JingStrozzi, MatteoHuang, JingfengStruzik, MichalHuang, QijinStrzałkowski, KarolHuang, Shu-ChiStrzemiecka, BeataHuang, ShuigenStrzhemechny, Yuri M.Huang, Yen-LinSTUERGA, DidierHuang, YuandingStuhr, UweHuang, ZhengmingStumpe, JoachimHuber, David L.Štverková, HanaHuber, TimStylianakis, MinasHudak, RadovanStyskalik, AlesHueso, Jose L.Su, An-ChungHughes-Riley, TheodoreSu, BoHuh, Do SungSu, JingHuh, Jung-BoSu, YingchaoHumar, MihaSuárez, FernandoHung, Kuo-YungSuárez, Oscar MarceloHung, Tai-FengSubramaniam, PrasadHunt, AdrianSuchanicz, JanHuo, DehongSuchorab, ZbigniewHuo, SuguoSudbrack, Chantal K.Hupa, LeenaSuder, WojciechHur, JaehyunSudhakar, VadirajaHurley, MikeSuematsu, KoichiHurst, RogerSugimoto, Koh-ichiHussain, BabarSugimura, HaruhikoHutchinson, BevisSugiura, YukiHutter, TanyaSuh, Jin-YooHvizdoš, PavolSui, TanHwang, Chi-SunSuib, StevenHwang, InchanSujova, AndreaHwang, Seung RimSuleria, HafizHwang, YongsungSullivan, EirinIandolo, DonataSumelka, WojciechIannazzo, DanielaSumets, MaximIatsunskyi, IgorSumi, ShoichiroIchikawa, YoSummerscales, JohnIconaru, SimonaSun, DaliIfelebuegu, AugustineSun, GuangyongIftekhar, SidraSun, HaoIglesias, EmiliaSun, HongyuIglesias-Juez, AnaSun, JingjingIglič, AlešSun, YingjieIgnaszak, AnnaSunasee, RajeshIgnat, MariaSunatsuki, YukinariIkeda, TaichiSunde, SveinIliopoulos, KonstantinosSunderland, PeterIljkić, DarioSunyol, Joan JosepIllán Gómez, Maria JoséSuplicz, AndrásIlles, Erzsebet Suragani Venu, LalithImai, HiroyukiSuraneni, PrannoyImanaka, MakotoSurmenev, Roman A.Imperatore, StefaniaSurreddi, Kumar BabuInada, RyojiSuryanto, BennyIncarnato, LoredanaSusic, MichaelInomata, KatsuhiroSusmel, SabinaInsepov, Zinetula (Zeke)Suzuki, JonInvernizzi, MartaSveda, MariaIonita, PetreSvetlík, JozefIordanskii, AlexeySvidró, József TamásIpsakis, DimitrisSweetman, MartinIrie, MasaoSwiatkowska-Warkocka, ZanetaIrukuvarghula, SandeepŚwiderski, WaldemarIsakov, DmitryŚwiercz, RafałIsanaka, Sriram PraneethŚwierczyńska, AleksandraIshikawa, RyoSwietoslawski, MichalIshikawa, YasuakiŚwiętosławski, MichałIslam, KamrulSycheva, G.A.Islamoglu, TimurSydlik, StefanieIsola, GaetanoSzala, MirosławIspas, AdrianaSzaller, ZsuzsannaIto, KotaSzalwinski, ChrisIto, MikioSzczepanik, BeataIto, TaichiSzczepanski, CarolineItoh, TamitakeSzcześ, AlexandraIvanda, MileSzczurek, AndrzejIvanova, T.Szegletes, ZsoltIwamoto, YujiSzekely, GyorgyIwanicki, AdamSzeląg, MaciejIwasa, TakashiSzeleszczuk, LukaszIwasaki, MasaokiSzepvolgyi, JanosIzu, NoriyaSzewczuk-Karpisz, KatarzynaIzzo, FrancescoSzewczyk, RomanJ. Rodríguez Trías, RafaelSzilágyi, IstvánJacak, JarosławSzilvia, KlébertJachalke, SvenSzkliniarz, AgnieszkaJackson, MarkSzlancsik, AttilaJackson, Mark J.Szolnoki, BeátaJackson, NathanSzostak, MarekJacob, AboudiSzwajka, KrzysztofJadczak, Joanna N.Szymanska-Chargot, M.Jadhav, Niteen G.Szymczak, TomaszJadwicienczak, WojciechSzymczyk, PatrycjaJafari, SajjadTabakovic, AmirJägle, EricTábi, TamásJahangir, IfatTabish, TanveerJaiswal, SwarnaTaboryski, Rafael J.Jajam, Kailash C.Tada, NaoyaJames, SagilTadanaga, KiyoharuJampaiah, DeshettiTadano, YuichiJamróz, PiotrTadmor, RafaelJamrozik, WojciechTadyszak, KrzysztofJamshidi, AliTaffa, Dereje HailuJan, MrázekTahara, HironobuJanakaloti Narayanareddy, Babu ReddyTaheri, HosseinJanakiraman, HarinarayananTahmoorian, FarzanehJanas, DawidTaiki, NakataJanczarek, MarcinTakada, TomoyaJänes, AlarTakaffoli, MahdiJang, Jae HoonTakagi, NoriakiJang, JinahTakagi, YasumasaJang, Keon-SooTakahashi, KazuakiJang, S.C. JasonTakahashi, KojiJang, Seung-YupTakahashi, YasuoJang, SoohwanTakahiro, KatsumiJang, Sung-HwanTakamatsu, SeiichiJangam, John Samuel DilipTakano, ToshiyukiJanicki, DamianTakasugi, TakayukiJanik, VitTakata, NaokiJanka, OliverTakayama, YoshiyukiJankauskaite, VirginijaTakayuki, BanJankovský, OndřejTakeda, YasunoriJankowska, AldonaTakeno, HiroyukiJanoš, PavelTakeoka, ShinjiJanovec, JozefTalaat, AhmedJanssen, GuidoTalapaneni, Siddulu NaiduJanusas, GiedriusTaleb, A.Jarvis, KarynTalebi, S. HesamodinJasielec, Jerzy J.Tallarico, MarcoJavaherdashti, RezaTalò, GiuseppeJayakumar, ArunkumarTalukdar, SudipJayamohan, HarikrishnanTamás, MankovitsJayatissa, Ahalapitiya H.Tamás, TörökJedrasiak, PatrykTamimi, SaeedJędrzejczyk, Roman J.Tamulevičienė, AstaJędrzejewska-Szczerska, MałgorzataTamura, KojiJedynak, KatarzynaTamura, RyoJeeyoung, YooTan, XianjunJeffs, SpencerTanaka, KazuoJelonek, KatarzynaTanaka, MasatoJelovica, JasminTanaka, TomonariJeng, Jiiang-HueiTanase, CorneliuJenkins, Michael G.Tang, Chao-WeiJenkins, Samir V.Tang, ChungangJennings, Jessica AmberTang, JieJeon, Ju-WonTański, TomaszJeong, Chang KyuTao, BingJeong, Dae-YongTao, ShuxiaJeong, Hoon EuiTapoglou, NikolaosJeong, Keun-YeongTappa, KarthikJeong, Mun SeokTarasov, Sergei YuJeong, Soon-JongTarbuk, AnitaJerby, EliTard, CédricJermakowicz-Bartkowiak, DorotaTardy, BlaiseJesson, DavidTaruta, SeiichiJezierski, JanTatar, JovanJeziorska, ReginaTataranni, PiergiorgioJha, MithileshTatarko, PeterJi, ShouxunTatsumi, YukiJi, VincentTattikota, Sudhir GopalJia, CharlesTatulian, Suren A.Jia, MingtuTatullo, marcoJia, RuTaupin, VincentJia, YuTavakoli, MahmoudJiang, JiangTavangarian, FariborzJiang, JingTavanti, FrancescoJiang, JunTavares, S.M.O.Jiang, RuinianTawfik, Sherif AbdulkaderJiang, WenpingTaylor, SpencerJiang, XiaoTcherdyntsev, Victor V.Jiang, XuchuanTeghil, RobertoJicsinszky, LászlóTehranchi, AmirhosseinJimenez Ballesta, Ana EvaTelegdi, JuditJimenez, CatalinaTen Kate, MelvinJimenez-Villacorta, FelixTeng, ChongJin, KailongTenza-Abril, Antonio JoséJin, SongwanTeo, Jeremy C.M.Jin, XinfangTeodorescu, FlorinaJinno, YoheiTeodoriu, CatalinJirasek, VitTeramoto, YoshikuniJirásková, YvonnaTerry, IanJirková, HanaTesfaye, FisehaJishkariani, DavitTesta, Maria LuisaJiu, JintingTestani, ClaudioJmerik, V. N.Testoni, NicolaJoana, Mesquita-GuimarãesTevetkov, NikolaiJohánek, ViktorTeymourian, HazhirJohanson, UrmasTeyssier, JeremieJohnson, ScooterThai, Duc-KienJones, J CliffThakur, GarimaJoos, JonasThakur, Vijay KumarJopek, HubertThang, Tran QuyetJordao, LuisaThapa, Prem S.Pastor, José L.Thapliyal, VivekJoshi, NiravkumarTheisen, WernerJoshi, SaumyaThiele, KlausJoshi, SurajThinon, EmmanuelleJozwik, JerzyThoelen, RonaldJuang, Jia-YangThomas, T.J.Juan-Valdes, AndresThrivikraman, GreeshmaJubeli, EmileThuault, AnthonyJugo, JosuThuillier, SandrineJu-Kim, YoungThünemann, AndreasJulien, Christian M.Tian, TianJun, Martin Byung-GukTibbetts, Katharine MooreJung, HaesungTibbitt, MarkJung, Hyun-DoTiemann, MichaelJung, In HwaTiferet, EitanJung, Jae-GilTimmel, KlausJung, Kyung-HyeTimofeev, Ivan V.Jung, SungpilTimoshkin, Alexey Y.Juodkazis, SauliusTiribocchi, AdrianoJurado-Sánchez, BeatrizTirovic, MarkoJürgen, BärTkach, AlexanderJüttner, SvenTkachenko, NikolaiJyothi, Rajesh KumarTkacz, J.Kaal, WilliamTlili, B.Kabanos, ThemistoklisToan, Nguyen VanKacem, NajibTochigi, EitaKacik, DanielToci, GuidoKaczmarek, BeataTodaro, LuigiKaczmarski, JakubTodhunter, LukeKada, SitaramaTohmura, Shin-ichiroKadimisetty, KarteekTokunaga, EijiKadokawa, Jun-ichiTolkou, AthanasiaKadyk, ThomasTolstoguzov, AlexanderKafexhiu, FevziTomanek, BoguslawKageshima, YosukeTomasz, DurejkoKairy, ShravanTomasz, MoscickiKaisarly, DaliaTomczyk, AdamKakar, Muhammad RafiqTomer, VijayKakiuchi, ToshifumiTomer, Vijay K.Kako, TetsuyaTomita, SatoshiKalácska, GáborTomków, JacekKalantar-Zadeh, KouroshTomlinson, DouglasKale, GirishTomota, YoKalfagiannis, NikolaosTomovic, MiletaKalina, LukášTomoyoshi, NishiumuraKallitsis, Joannis K.Tomšič, BrigitaKalmár, JózsefTonacci, AlessandroKalogirou, OrestisToncelli, AlessandraKalsi, SwarnTondi, GianlucaKalteremidou, Kalliopi-ArtemiTonelli, Alan EdwardKamali, SaeedTonelli, MonicaKamat, AmarTong, MingmingKamdem, Donatien PascalTorgal, F.PachecoKamegawa, TakashiTorić, NenoKamel, KarolTornabene, FrancescoKamiński, MarcinToro, Roberta G.Kaminski, PiotrTorre, Maria LuisaKamitaka, YujiTorrens-Serra, JoanKamperidou, VasilikiTorres Lagares, DanielKampitsis, AndreasTorres Vaamonde, Jose EnriqueKan, Chi-waiTorres-Carrasco, ManuelKanari, NdueTorroba, TomásKanavillil, NandakumarTortschanoff, AndreasKandola, Baljinder KaurTorun, HamdiKaneko, KenjiToschi, StefaniaKaneko, YoshihisaTosello, GuidoKanematsu, HideyukiTotaro, GraziaKang, Chung GilToth, LaszloKang, Do HyunToyao, TakashiKang, HyoTraini, ToninoKang, Hyun WookTrainiti, GiuseppeKang, JihoonTran, FabienKang, Min GyuTranell, GabriellaKang, PilgyuTravieso Rodríguez, J. AntonioKang, Seong JunTretiakov, KonstantinKang, Sung-HoonTretsiakova-McNally, SvetlanaKang, XinTricase, CaterinaKang, YCTrifol, JonKangas, MichaelTrinh, ThuatKanno, TakahiroTripathi, Jitendra KumarKantartzis, Nikolaos V.Tripathi, KumudKao, Nien-HsinTripodi, GianlucaKapcia, KonradTrivedi, RahulKaplar, RobertTrivellin, NicolaKapłonek, WojciechTrochu, FrançoisKappagantula, KeertiTrögl, JosefKappera, RajeshTrojanová, ZuzankaKapsalis, Vasilios C.Trotta, GianlucaKapsalis, VassilisTrško, LiborKar, SupratikTrukhanov, Alex VKara De Maeijer, PatriciaTrukhanov, SergeyKaragiozova, DoraTruong, Vi KhanhKarahan Toprakci, Hatice AylinTrusso Sfrazzetto, GiuseppeKarakalas, AnargyrosTruster, TimothyKarakasidis, T.E.Trzepieciński, TomaszKaralekas, DimitrisTrzop, ElzbietaKarapanagiotis, IoannisTsai, Ang-ChenKarazhanov, SmagulTsai, Chia-lungKarbalaei Baba, AlirezaTsangouri, EleniKarbowniczek, JoannaTsao, Lung-ChuanKareiva, AivarasTsay, CHien-YieKarg, Michael Cornelius HermannTschierske, CarstenKargl, RupertTsitsilianis, ConstantinosKarnaouri, AnthiTsoi, JamesKarnati, PriyankaTsubaki, ShuntaroKarolczuk, AleksanderTsutaoka, TakanoriKarolus, MalgorzataTsuyumoto, IsaoKarpov, SergeyTsvetanov, ChristoKarpukhina, NataliaTsvetkov, NikolaiKartopu, GirayTsvetkov, PavelKartsonakis, IoannisTudorache, FlorinKaščák, ĽubošTudoroiu, NicolaeKaseem, MosabTulliani, Jean-MarcKasem, Kasem K.Tulunoglu, IbrahimKashani, AliTumarkin, Andrey V.Kashi, SimaTunesi, MartaKashimura, KeiichiroTung, Tran ThanhKashiwagi, ShinichiroTuominen, JariKassanos, PanagiotisTuovinen, OlliKataoka, YusukeTurco, AntonioKatarzyna, NiemirowiczTurco, EmilioKate, KunalTurco, GianlucaKateris, DimitriosTurkmen, MustafaKatin, Konstantin P.Turkowski, VolodymyrKatoh, KenjiTurner, RichardKatsounaros, IoannisTuron, PauKattoura, MichealTussupzhanov, AidynKatunin, AndrzejTutunaru, BogdanKatz, Jonathan I.Tverdokhlebov, S. I.Kaufman, Michael J.Tylkowski, BartoszKaunas, Roland R.Tytła, MalwinaKavita, KumariTzounis, LazarosKawabata, YuichiroUan, Jun-YenKawai, ToshiyukiUcciardello, NadiaKawakami, HiroshiUchida, YoshiakiKawalec, Magdalenaüçüncü, MuhammedKawamura, IzuruUdalov, Oleg GeorgievichKazak, AndreyUddin, AshrafKazda, TomášUeda, TaroKazek-Kęsik, AlicjaUeji, RintaroKažys, Rymantas JonasUeshima, NobufumiKeist, Jayme S.Ugrina, MarinKelder, Erik M.Uhlig, JensKeller, BrandisUhlig, ThomasKeller, ClémentUjihara, MasakiKellesarian, SergioUji-i, HiroshiKemmegne-Mbouguen, Justin ClaudeUklejewski, RyszardKennedy, JohnUkrainczyk, NevenKeppler, JuliaUllrich, CarstenKern, WolfgangUmehara, NoritsuguKeršner, ZbyněkUmer, RehanKertèsz, SzabolcsUnal, ResitKesavan, R.Uneyama, TakashiKeshavarz Hedayati, MehdiUnterlass, Miriam M.Kessel, DavidUnterweger, ChristophKessler, SylviaUpare, Pravin P.Ketov, SergeyUr, Soon-ChulKettwich, Sharon C.Urban, PetrKevadiya, BhaveshUrbani, MaxenceKevern, JohnUrbicain, GorkaKhabaz, FardinUrbonas, LiudvikasKhadka, Dhruba B.Usher, Tedi-MarieKhairullina, VeronikaUshida, AkiomiKhakalo, SergeiUshiyama, HiroshiKhaliq, JibranUsui, HidetomoKhan, ArafatUtgikar, VivekKhan, AurangzebUtton, ClaireKhan, Mohammad AshrafUzunlar, ErdalKhan, MuhammadUzunova, Ellie L.Khandaker, MorshedVaccari, AngeloKhanna, RohitVahabi, HamedKhansur, Neamul HayetVahabi, HenriKhare, SanjayValange, SabineKharel, ParashuValavanides, Marios S.Kharin, ViktorValdemar Fernandes, JoséKharkar, PrathameshVǎleanu, Mihaela CristinaKhatib, JamalValentini, FedericaKhatibi, G.Vallés-Lluch, A.Khattab, MuhammadVallianatos, FilipposKheradmand, NoushaValov, IliaKhirallah, KareemValvano, StefanoKholodnaya, GalinaValvo, Paolo S.Khosravani, Mohammad RezaVamanu, EmanuelKhung, Yit LungVan Der Beek, CornelisKhurshid, ZohaibVan Driel, W.D. (Willem)Kichambare, PadmakarVan Driessche, AlexanderKiciński, WojciechVan Herwijnen, Hendrikus W. G.Kiefer, RudolfVan Noort, DannyKieliszek, MarekVan Noort, ReinierKieninger, JochenVancaeyzeele, CédricKierys, AgnieszkaVanmeensel, KimKiesewetter, DmitryVaqueiro, PazKihara, NobuhiroVarga, ZoltanKihara, Shin-ichiVarghese Chacko, JenuKik, TomaszVas, LászlóKikuchi, HiroakiVasandani, PareshKilmametov, AskarVasconcelos, Helena CristinaKim, Chan-JungVasile, CorneliaKim, DaehwanVasile, SimonaKim, Dai-SikVassiliadis, SavvasKim, DonginVautard, FredericKim, Dong-JooVaz, CarlosKim, Felix SunjooVaz, Irene PinaKim, Gue-hyunVazquez Besteiro, LucasKim, GyuyongVazquez Martinez, Juan ManuelKim, HeungsooVázquez, ManuelKim, HojoongVedarajan, RamanKim, HongsukVega Gutierrez, SarathKim, Hyun TakVejux, AnneKim, Hyun-SukVekinis, GeorgeKim, HyunsungVelasco López, Francisco JavierKim, Il TaeVelasco-Vélez, Juan JesúsKim, InsooVelazquez-Perez, Jesus EKim, JaehyunVELEA, Marian NicolaeKim, JanghoVelkavrh, IgorKIM, Jeong-JooVelliyagounder, KabilanKim, Ji SikVemula, HarikaKim, Jong HyunVendik, IRINAKim, Jong-HoonVenditti, AlessandroKim, Jong-KiVenditti, IoleKim, JongseongVendrell, XavierKim, JooyounVenetis, JohnKim, JungGuVenezia, Anna MariaKim, Jung-GuVenkata, Kiranmayi AbburiKim, KyunghoonVenkatesan, JayachandranKim, KyungmokVereshchagin, Oleg S.Kim, MinJoongVereshchaka, Alexey A.Kim, Mun-KyeomVergara, DiegoKim, Myung JunVerlinden, Jouke C.Kim, Nam KyeunVerma, Madan LalKim, Sang MoonVerma, RanjitKim, Sang-ilVerma, SannyKim, SangtaeVernardou, DimitraKim, SeokminVerre, SalvatoreKim, Seong-GonVerstraete, KevinKim, Seung-JooVertruyen, BenedicteKim, SeyoungVesel, AlenkaKim, Si JoonVezzu, DileepKim, Soo YoungVicente, Miguel A.Kim, Su-HyeonVicenzo, AntonelloKim, Tae-HyungVidal, HilarioKim, Yoon YoungVidor, Fábio F.Kim, Young SikVieira, Manuel FernandoKim, YounghunViespe, CristianKim, Young-MinVijayan Sobhana, DilimonKim, Yu KwonVijayavenkataraman, SanjairajKim, Yun YoungVikas, VisheshKimura, MunehiroVila, AnnaKinoshita, TakumiVila-Comamala, JoanKinuthia, JohnVila-Vilela, Jose LuisKioseoglou, GeorgeVilcakova, JarmilaKiparissides, CostasVilhena, LuísKirane, KedarVilla, ElenaKirillova, AlinaVillanneau, RichardKirste, RonnyVillecco, FrancescoKiryukhantsev-Korneev, Ph.V.Vincent, DidierKishimoto, HirotatsuVincent, ThomasKisielewicz, TomaszVinci, AntonioKiss, JánosVinogradov, SergeyKitagawa, JiroViola, FabioKitahama, YasutakaVioque, IgnacioKitahara, TatsumiVisan, TeodorKitajou, AyukoVisaveliya, NikunjkumarKitamura, ChiakiVishnyakov, AlekseyKitamura, MichihideVisintin, PhillipKitazono, KoichiViskadourakis, Zacharias A.Kityk, IwanViswanadam, GouthamKiwa, ToshihikoViswanathan, VaishakKlapötke, Thomas M.Viter, RomanKlein, Lisa C.Vithana, ChamindraKlein, RolandVitry, PaulineKlemm, Richard A.Vittadini, AndreaKlimach, HaraldVittorio, DiCoccoKlimczyk, PiotrViva, Federico A.Klimek, KatarzynaViviani, MassimoKlimeš, LubomírVivo, PaolaKlimkowicz-Pawlas, AgnieszkaVizureanu, PetricǎKlontzas, MichaelVlad, StolojanKlose, ChristianVleugels, Jozef (Jef)Klotz, MichaelaVlieg, EliasKnapek, MichalVo, Duc-ThangKnite, MārisVohra, VarunKnop, YanivVoliani, ValerioKnowles, AlexanderVolker, KörstgensKnyazeva, MarinaVollbrecht, JoachimKo, Jang MyounVolmer, MariusKo, Myoung-SooVolochaev, V. A.Ko, Won-SeokVolpe, MaurizioKo, Young GunVon Bardeleben, H.J.Kobayashi, MakikoVora, AnkitKoblischka, Michael RudolfVorotyntsev, IlyaKochmański, PawełVosch, TomKodera, ToshiroVougioukas, EmmanouilKoduru, Janardhan ReddyVourdas, NikolaosKodzius, RimantasVourkas, IoannisKoettgen, JuliusVourna, PolyxeniKogia, MariaVoyiadjis, George Z.Koh, Young-HagVrabič Brodnjak, UrškaKöhler, KarstenVrana, NihalKohlmann, HolgerVranceanu, Diana M.Koinkar, PankajVrancken, BeyKoirala, NiranjanVrsaljko, DomagojKoivuluoto, HeliVu Bac, NamKoizumi, HiroyasuVukelic, GoranKojima, KenichiVul, AlexanderKojima, ToshikatsuVuong, Thu V.Kojima, YoshiyukiVuye, CedricKokado, KentaWachowski, MarcinKokanyan, NinelWachs, IsraelKokkoris, GeorgeWada, YujiKolan, KrishnaWadati, HirokiKolanowski, Jacek L.Wahab, M. FarooqKolaric, BrankoWainstein, DmitryKolasinski, Kurt W.Waite, Matthew M.Kolcunová, IraidaWakai, FumihiroKolmas, JoannaWakimoto, HiroakiKoltunowicz, TomaszWałęsa-Chorab, MonikaKomasa, SatoshiWalker, JulianKombaiah, BoopathyWalkley, BrantKomljenovic, DraganWalock, MichaelKonar, ArkaprabhaWalsh, William R.Konda Gokuldoss, PrashanthWalther, FrankKondinski, AleksandarWan, ChaoyingKondo, MioWang, BinghaoKonegger, ThomasWang, ChenKong, DemingWang, DaweiKönigsberger, MarkusWang, GaoxueKononova, Svetlana V.Wang, JiyeKonshina, ElenaWang, JunjieKontic, RomanWang, LizhuKontonasaki, EleanaWang, LuKontoni, Denise-PenelopeWang, PaiKontou, EviWang, PangpangKontturi, EeroWang, PengtaoKonyashin, IgorWang, Peng-Yuan (George)Koohbor, BehradWang, QingQingKootala, SujitWang, QinyiKooyman, Patricia J.Wang, RuofanKopal, IvanWang, ShuodaoKopeček, JaromírWang, XiKoplík, JanWang, XiaofengKoplin, ChristofWang, XiaohongKoponen, Antti IlmariWang, Xiao-MingKopyściański, MateuszWang, Xiao-YongKordatos, EvangeloWang, YingKoren, KlausWang, YongxiangKoric, SeidWang, YuKORIN, ELIWang, ZhiguangKorivi, NagaWang, ZhongchangKornél, MájlingerWangler, Timothy P.Korosteleva, Elena N.Wan-Wendner, RomanKorsunsky, Alexander M.Wardeh, GeorgeKoruza, JurijWark, MichaelKorzeniewska, EwaWarmuzek, MalgorzataKositski, RomanWarner, MarkKostas, Emily TWaryoba, Daudi R.Kostryzhev, Andrii G.Watanabe, FumiyaKosuke, NozakiWatanabe, IkumuKosuke, SugawaWatanabe, MasahitoKotek, JanWatras, AdamKotoka, RubenWatzky, MurielleKotov, AntonWebb, R. ChadKotsiomiti, EleniWeber, BentKotsuchibashi, YoheiWeber, FranzKoukolikova, MartinaWeber, JulianeKoumoulis, DimitriosWebster, John G.Koumoulos, Elias P.Weems, Andrew C.Kourousis, KyriakosWehner, MartinKourousis, Kyriakos I.Wei, DongbinKoutny, DanielWei, GangKoutzarova, TatyanaWei, HuaKovács, ZsoltWei, KayaKovalcikova, AlexandraWei, ZhishunKovalevsky, Andrei V.Weigand, AnnikaKovářík, OndřejWeigel, RobertKowal, JuliaWeinberger, ChristianKowalak, StanisławWeise, JörgKowalczuk, MarekWeiß, SabineKowalczuk, Marek M.Weisz-Patrault, DanielKowalczyk-Gajewska, KatarzynaWeitkamp, TimmKowalska, EwaWells, BarrettKowalski, AleksanderWenisch, SabineKowalski, KarolWertz, Philip W.Kowalski, LukaszWestover, Andrew SKozempel, JánWeydts, TristanKozlova, Ekaterina A.White, MarkKozlowska, JustynaWhite, Mary AnneKozłowska, MariolaWhitmer, JonathanKozyukhin, Sergey A.Whittaker, Mark T.Kraft, Reuben H.Whittaker-Brooks, LuisaKrahl, ThoralfWibowo, DavidKrahne, RomanWickramaratne, DarshanaKraker, ElkeWidbiller, MatthiasKrämer, LisaWidenmeyer, MarcKrasnoslobodtsev, Alexey V.Wieckiewicz, MieszkoKrause, Hans-JoachimWierzbicka-Miernik, AnnaKrebs, MelissaWiesbrock, FrankKregiel, DorotaWieslawa, Nocun-WczelikKren, VladimirWilding, MartinKrenner, HubertWilhelm, ReneKressler, JoergWilkinson, AngusKrim, JacquelineWillander, MagnusKrisyuk, VladislavWille, KayKrivtsov, IgorWille, SebastianKřivý, VítWilliams, Christopher BryantKrohns, StephanWilliams, E.Król, MagdalenaWilsens, KarelKrolczyk, GrzegorzWilson, ClaudiaKrólczyk, GrzegorzWilson, Glenn V.Królczyk, JolantaWinczek, JerzyKrólicka, AgnieszkaWinczewski, SzymonKroll, PeterWiniarski, JuliuszKrstulović-Opara, LovreWinkler, HeinzKrumeich, FrankWinter, Benjamin D.Kruszynski, RafalWisniewski, AdamKrymsky, Valeriy V.Wiśniewski, MarekKryschi, CarolaWitkowski, ArturKrystofiak, TomaszWitt, KatarzynaKrzanowski, James E.Wittmann, FolkerKrztoń-Maziopa, AnnaWłodarczyk, Paweł P.Ksiazek, MarzannaWlodarczyk, RenataKtena, AphroditeWoeltje, MichaelKu, Bon-CheolWojciech, BorekKuang, WenjunWojciechowski, ŁukaszKubiak, TomaszWójcik, GrzegorzKubik, ZdenekWojewoda-Budka, JoannaKubina, RobertWolfe, Tonya B.Kubo, MasatakaWolloch, MichaelKubzova, MonikaWolny, StefanKučera, MariánWondraczek, KatrinKucerova, LudmilaWong, EdgarKuchariková, LenkaWong, EugeneKuchmizhak, AleksandrWongkasem, NantakanKuciej, MichałWoodward, Robert T.Kucinska-Lipka, JustynaWorkman, VictoriaKucuk, IsrafilWoszuk, AgnieszkaKudelski, AndrzejWoyciechowski, PiotrKudiiarov, ViktorWright, JonKudlek, EdytaWróbel, JanKudryashov, Sergey I.Wróbel, MirosławKugler, SzymonWróblewski, GrzegorzKujala, SamiWu, Jyh-HorngKujan, OmarWu, LiliKujawa, JoannaWu, MenghuaiKujawski, WojciechWu, QiKukla, ChristianWu, R. J.Kukli, KaupoWu, ShenghuaKulagin, RomanWu, TaoKulichikhin, ValeryWu, XiangfaKulinich, SergeiWu, XijiaKulka, JozefWuttke, StefanKulkov, Sergei N.Wycisk, RyszardKulshreshtha, PrashantWyman, IanKumal, RajuWyszyński, DominikKumanek, BogumiłaXavier, José Manuel CardosoKumar Mishra, YogendraXi, WeixianKumar, AmitXia, GuanglinKumar, DhananjayXia, TianKumar, ManishXiao, YangKumar, NarendraXie, HaiboKumar, RajXin, HaiKumar, SandeepXu, JieKumatani, AkichikaXu, LuKumeria, TusharXu, TaoKunčická, LenkaXu, XiaoxueKundin, JuliaXu, Yi-JunKundu, JoydipXu, YongKundu, ManabXue, JinkaiKunimine, TakahiroXue, XingjianKunioka, MasaoYadav, SushmaKunjukunju, SangeethaYadavalli, TejabhiramKuo, Wen-TenYadgarov, LenaKurc, BeataYagüe-Fabra, José AntonioKurcok, PiotrYamabe, JunichiroKurda, RawazYamaguchi, MasahikoKuryntsev, Sergey VyacheslavovichYamaguchi, SatoshiKusiak-Nejman, EwelinaYamaguchi, SeijiKustov, SergeyYamaguchi, TetsuoKuzhir, PolinaYamakov, Vesselin I.Kuznetsov, SergeyYamamoto, GoKuznetsov, Vladimir E.Yamane, HisanoriKuznetsova, IrenYamashita, YoichiKuźnicka, BogumiłaYan, DayunKvasha, AndriyYan, LiangKwapinska, MarzenaYan, LiboKwiatkowski, MiroslawYan, NingKwiecień, IwonaYanagida, SayakaKwon, Hyuck-InYanase, KeijiKwon, Jae-SungYanase, TakashiKwon, Oh SeokYanaseko, TetsuroKwon, Seong JungYang, Beom JooKwon, Soon-HongYang, ChengKwon, Tae-YubYang, Chih-TsungKwon, Young-WanYang, Chung-WeiKyeongsik, WooYang, LeiKyle, StuartYang, Rong-SenKylian, OndřejYang, ShaoweiKyratsi, TheodoraYang, ShoufengKyratsis, PanagiotisYang, SungchulKyzas, GeorgeYang, Teng-ChunKyzioł, KarolYang, Tian-ZhiLa Mantia, Francesco PaoloYang, Tzu-SenLa Rosa, GuidoYang, WeiLa Spada, LuigiYang, XiaoyunLabisz, KrzysztofYang, YingchaoLabram, JohnYano, MitsuakiLacava, CosimoYao, HuiLacey, MatthewYao, TiankaiLadak, SamYapici, Murat KayaLafalce, EvanYasin, SohailLagos, Miguel ÁngelYasnikov, IgorLagrow, Alec P.Yasui, KyuichiLähnemann, JonasYasui, ShintaroLahouij, ImèneYates, JonathanLai, Wen-Fu ThomasYates, Matthew Z.Lai, YuekunYavuz, A. KadirLaiarinandrasana, LucienYazdani Sarvestani, HamidrezaLaitala, KirsiYe, HuaiyuLajeunesse, Dennis RichardYe, JongpilLakshminarayana, PolavarapuYe, LongLamberti, LucianoYe, YanqiLambiase, FrancescoYe, YuLamíkiz, AitzolYe, ZhaoyangLampakis, DimitriosYeager, JohnLampke, ThomasYeh, Chun-LiangLanceros, SenentxuYeih, Wei-ChungLand, MartinYentekakis, Ioannis V.Lange, HeikoYeo, In-SungLanger, Jerzy JYeo, JunyeobLantsoght, Eva Olivia LeontienYeom, JunseokLAO, JonathanYeom, TaihoLao, Ka UnYeon, Jung HeumLapington, JonYigang, YanLaptev, AlexanderYildirim, HalidLarkiola, JariYilmaz, MehmetLaroche, GaétanYim, TaeeunLaromaine Sagué, AnnaYin, WuliangLarosa, ClaudioYin, XiaodongLarouche, DanielYing, HanzeLarraneta Landa, EnekoYip, Kay-pongLarsson, KarinYliniemi, JuhoLashgari, Hamid R.Yo, TomotaLässig, Torsten R.Yokoi, TatsuyaLastovina, TatianaYoo, ByungseokŁatka, LeszekYoo, Chung-YulLaura, MorettiYoo, Dong JinLaurent, CharpinYoo, DongwonLaureti, StefanoYoon, HyeonseokLauria, AlessandroYoon, Jeoung SeokLaurich, BenYoon, Ji-WookLaurila, TomiYoon, Keun-ByoungLautner, GergelyYoon, Moon-YoungLaverty, GarryYoon, SonghunLawler, JennyYoshida, HidehiroLázár, IstvánYoshida, KengoLazarus, NathanYoshida, SanichiroLazurenko, Daria VYoshihara, HiroshiLazzara, GiuseppeYoshitake, HideakiLazzeri, RobertaYou, Chun-YeolLe Coq, DavidYou, Tae-SooLe Febvrier, ArnaudYou, Won-heeLe, DuyYoung, Cheng FuLeal, RuiYoung, SimonLebeau, BenedicteYounis, AdnanLebon, FrédéricYousif, BelalLebyodkin, M. A.Youssry, MohamedLeccese, FabioYperman, JanLecomte, PhilippeYu, HakkiLederer, Franziska L.Yu, Hsin HerLedesma, Enrique FernandezYu, Ing-SongLee, ByeongchanYu, JianLee, Byung ChulYu, JiayiLee, Chang-HoonYu, Rena C.Lee, Cheng-IYu, Seung-HoLee, Chi HYu, TaekyungLee, Chia-HungYu, Tzyy LeonLee, Chih-HaoYu, YunLee, Chul-HeeYurchenko, NikitaLee, Czang-HoYusa, Shin-ichiLee, Eon SooYusenko, Kirill V.Lee, GyoZacharatos, FilimonLee, H. W.Zafar, MuhammadLee, Hung BinZagórski, IreneuszLee, IlkeunZagoruiko, AndreyLee, JaehyunZagula-Yavorska, MaryanaLee, Jin-KyunZahid, MalihaLee, Jong-SookZahiri Azar, MaryamLee, JoonheeZaidi, AliLee, Jun SeokZaitsev, Alexandr A.Lee, Kee SungZakinyan, ArthurLee, Kyung-JinZaleski, RadosławLee, Li-TingZallo, EugenioLee, Sang-KonZambon, DanielLee, Seung HeeŻamojć, KrzysztofLee, Seung-JungZangari, GiovanniLee, Sung-MinZaniewski, John P.Lee, SunwooZannotti, MarcoLee, WonohZanotto, FedericaLee, WookjinZaplotnik, RokLee, Young-ChulZappia, StefaniaLee, Young-ilZarepoor, MasoudLee, YoungkwanZarkadoulas, AthanasiosLee, YousubZayachuk, YevhenLeem, Jung WooZazpe, RaulLeevy, W. MatthewZdarta, JakubLefebvre, FredericZea Escamilla, EdwinLéger, BastienZębala, WojciechLeger, LilianeZechel, StefanLehnherr, DanZekker, IvarLei, MeiZekonyte, JurgitaLeinenbach, ChristianZeleny, ReinhardLeitner, MartinZeng, HulieLeiviskä, KaukoZeugolis, DimitriosLeliaert, JonathanZhang, AnqiLemeune, AllaZhang, ChaoLemu, Hirpa G.Zhang, DouLeonardi, Salvatore GianlucaZhang, FanLeonelli, CristinaZhang, JianqiangLeonowicz, MarcinZhang, JulieLeopold, ChristianZhang, LaichangLepidi, MarcoZhang, LiangliangLeporatti, StefanoZhang, LijunLeprince, YaminZhang, MengLerche, DietmarZhang, NanLerga, JonatanZhang, RuiyongLesar, BoštjanZhang, RunLesiak, PiotrZhang, ShuaifangLesiuk, GrzegorzZhang, WeiLesz, SabinaZhang, WenxuLeszczyńska-Madej, BeataZhang, WuLetcher, ToddZhang, WujieLeung, Siu N.Zhang, XiaodanLevchenko, IgorZhang, XuefengLewandowska, JolantaZhang, Xueqiang AlexLeyva-Pérez, AntonioZhang, YijieLezzerini, MarcoZhang, YuLgaz, HassaneZhang, ZheLi, DuoZhao, ChumingLi, HaijunZhao, Evan WenboLi, JichaoZhao, HanLi, JiehuaZhao, HongLi, JunZhao, LianfengLi, LiZhao, LiJiaLi, LinZhao, ShanLi, LingweiZhao, XinLi, MingxingZhao, XinxinLi, RuosongZhao, YajunLi, ShanshanZhao, YiliangLi, ShiqiZhao, ZongminLi, StevenZheng, KaiLi, Tomi T.Zheng, KailunLi, WeidongZheng, LiqiuLi, XiaojunZheng, XintingLi, XiaomingZherebtsov, SergeyLi, YiZhilyaev, AlexanderLi, YingZhitova, Elena S.Li, YiwenZhong, TuhuaLi, YiyanZhong, XiangyuLiagre, BertrandZhou, ChiLian, JunheZhou, HongLiang, YunhongZhou, J.Liao, Shu-ChuanZhou, QiLiaw, Chya-YanZhou, YouLichtenegger, HelgaZhou, YuanshengLignola, Gian PieroZhou, YuqingLigon, Samuel ClarkZhou, ZhiLiguori, BarbaraZhu, BinLikodimos, VlassisZhu, DonghuiLim, Chung-SunZhu, LiangdongLim, Dae-WoonZhu, WeiLim, Dong ChanZhu, XiangweiLim, Eun-KyungZhukovskyi, MaksymLim, JoonwonZhuravlev, VictorLim, KhoonZiąbka, MagdalenaLim, Mary Lyn C.Zidki, TomerLima, CarmineZiegler-Borowska, MartaLin, Dan-JaeZielinska, BeataLin, JunrenZielińska-Jurek, AnnaLin, Pai-ChenZienert, TiloLin, Wei-ChihZimbardi, FrancescoLin, WenyeZinna, FrancescoLin, XiaojieZiora, ZytaLin, Yang-weiZiouche, KatirLin, Yuan-HuaZitoune, RedouaneLin, ZekaiZkria, AbdelrahmanLindberg, GabriellaŻmudzki, JarosławLindner, MartinaZocca, AndreaLinert, WolfgangZografopoulos, Dimitrios C.Ling, MauriceZoidis, PanagiotisLing, MengZok, Frank W.Linhardt, PaulZoppellaro, GiorgioLinul, EmanoilZornoza, EmilioLionetto, FrancescaZozulya, AlexeyLiotier, Pierre-JacquesZucchi, FabrizioLiotta, LeonardaZuhlke, Craig A.Lipiec, PiotrZukowski, WitoldLipski, AdamZuo, FeiLisi, LucianaZupančič, MatevžLisiecki, AleksanderŽuperl, UrošLisnevskaya, I.V.Zur, Krzysztof KamilLisovenko, DmitryZych, TeresaLiu, Cheuk LunZyła, Gaweł

